# Hierarchical self-assembly of a reflectin-derived peptide

**DOI:** 10.3389/fchem.2023.1267563

**Published:** 2023-09-21

**Authors:** Ana Margarida Gonçalves Carvalho Dias, Inês Pimentel Moreira, Iana Lychko, Cátia Lopes Soares, Arianna Nurrito, Arménio Jorge Moura Barbosa, Viviane Lutz-Bueno, Raffaele Mezzenga, Ana Luísa Carvalho, Ana Sofia Pina, Ana Cecília Afonso Roque

**Affiliations:** ^1^ Associate Laboratory i4HB—Institute for Health and Bioeconomy, School of Science and Technology, Universidade NOVA de Lisboa, Caparica, Portugal; ^2^ UCIBIO—Applied Molecular Biosciences Unit, Department of Chemistry, School of Science and Technology, Universidade NOVA de Lisboa, Caparica, Portugal; ^3^ Department of Health Sciences and Technology, ETH Zürich, Zürich, Switzerland; ^4^ Paul Scherrer Institute, Villigen, Switzerland

**Keywords:** supramolecular, self-assembly, peptides, reflectins, hydrogels, films, optical materials, bio-based materials

## Abstract

Reflectins are a family of intrinsically disordered proteins involved in cephalopod camouflage, making them an interesting source for bioinspired optical materials. Understanding reflectin assembly into higher-order structures by standard biophysical methods enables the rational design of new materials, but it is difficult due to their low solubility. To address this challenge, we aim to understand the molecular self-assembly mechanism of reflectin’s basic unit—the protopeptide sequence YMDMSGYQ—as a means to understand reflectin’s assembly phenomena. Protopeptide self-assembly was triggered by different environmental cues, yielding supramolecular hydrogels, and characterized by experimental and theoretical methods. Protopeptide films were also prepared to assess optical properties. Our results support the hypothesis for the protopeptide aggregation model at an atomistic level, led by hydrophilic and hydrophobic interactions mediated by tyrosine residues. Protopeptide-derived films were optically active, presenting diffuse reflectance in the visible region of the light spectrum. Hence, these results contribute to a better understanding of the protopeptide structural assembly, crucial for the design of peptide- and reflectin-based functional materials.

## 1 Introduction

Cephalopods possess incredible protein variability at the structural level (Tan et al., 2015). Reflectins, in particular, are a family of structural proteins responsible for cephalopod camouflage upon environmental stress (Crookes et al., 2004). They organize into platelets in specialized skin cells, namely, iridophores, that act as biological Bragg reflectors, and leucophores, that act as light scatterers (Levenson et al., 2017). Over the last years, the recombinant production of reflectins revealed their technological potential as photonic and electronic bio-based materials (Fudouzi, 2011; Ordinario et al., 2014). However, for the design of reflective bio-inspired advanced functional materials, a better understanding of the sequence–structure–function relationship is required.

Reflectins are intrinsically disordered proteins composed of highly conserved and repetitive domains (called “repeating domains”) rich in hydrophobic, charged, and polar residues (Crookes et al., 2004), which are interspersed by variable linkers. Reflectins are hydrophobic and prone to aggregation, causing them to be highly insoluble and difficult to characterize by common biophysical methods. This has been circumvented by studying reflectins in solutions that promote their dissolution or by studying truncated or engineered reflectin sequences that can be easily solubilized in aqueous environments [e.g., Domain 2C and Ref(2C)4 (Dennis et al., 2017; Xu et al., 2021), RefCBA (Qin et al., 2012), D1 domain (Guan et al., 2017), and RfA1TV (Umerani et al., 2020)].

**FIGURE 1:**
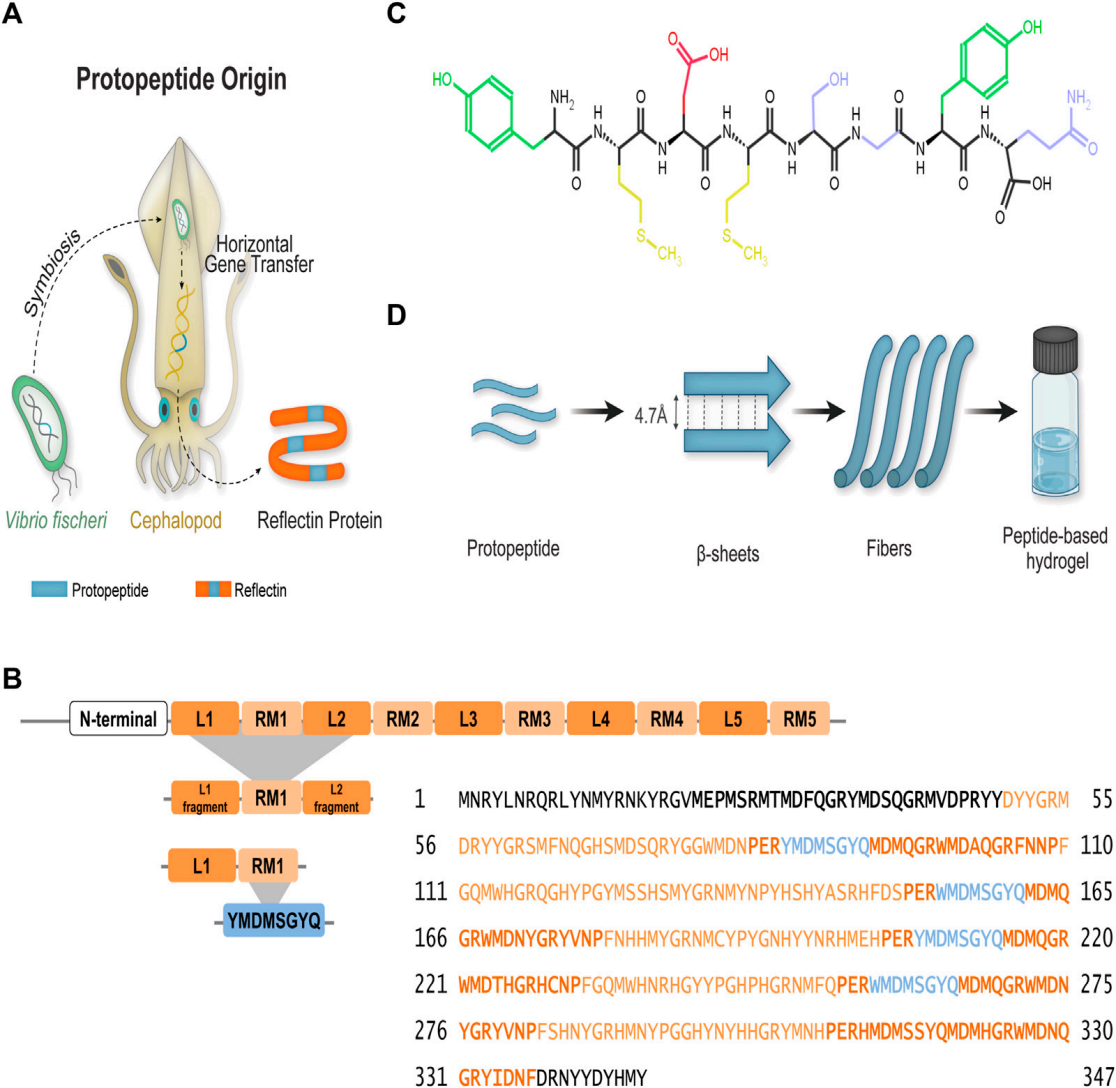
Protopeptide—the basic unit of **r**eflectins. **(A)** Proposed origin of the basic unit of reflectins—the protopeptide. The symbiosis between a bacterium *Vibrio fischeri* and cephalopods resulted in a gene transfer that led to the formation of the reflectin gene. The expressed reflectin protein incorporates several copies of the protopeptide (blue). **(B)** Reflectin sequences are composed by a highly conserved N-terminal sequence (bold) and several repeating motifs (orange) connected by linkers (dark orange). Several truncated sequences have been generated to study reflectin’s self-assembly, with *D. pealeii* reflectin A1 (Uniprot: D3UA43) being one of the most studied sequences, and the classification of repeating motifs and linkers was adapted from Umerani et al. (2020). Repeating motifs contain the protopeptide sequence or its variants (blue): (Y/W/G)MD(M/F) X(G/N)X_2_, (X = S, Y, Q, W, H, R), adapted from Guan et al. (2017). **(C)** Protopeptide chemical structure YMDMSGYQ used in this work, with highlighted amino acid side chains in different colors—Tyr (green), Met (yellow), Asp (red), and Gln, Gly, and Ser (blue). **(D)** The distinct hierarchical levels approached in this work to understand protopeptide self-assembly into a macroscopic hydrogel.

Guan and colleagues studied in parallel the self-assembly of a full reflectin sequence (SoRef2 from cuttlefish with four D domains and 242 amino acids), one repeating domain of a such sequence with fragments of N- and C- terminal linkers (D1 domain and 44 amino acids), and the protopeptide (eight amino acids) found in triplicate in each D domain. The YMDMSGYQ sequence—or its variants (Y/W/G)MD(M/F) X(G/N)X_2_, (X = S, Y, Q, W, H, R) flanked by two tyrosine–phenylalanine-rich (YF-rich) regions—was identified as a protopeptide derived from *Vibrio fishery* bacteria during their symbiotic partnership with cephalopods (Figures 1A,B) (Guan et al., 2017). These studies were performed in the presence of detergent (sodium dodecyl sulfate, SDS) and aromatic compounds, such as imidazole. SDS was found to solubilize and stabilize the protein while hindering ionic strength effects. Furthermore, it was postulated that the imidazolium ring from imidazole promotes π–π stacking interactions with tyrosine aromatic rings, favoring the self-assembly of reflectin and derived peptides into higher-order nanostructures (Guan et al., 2017). In fact, it is reported that imidazole-based molecules establish π–π interactions through the aromatic rings, and they can assemble into supramolecular structures using π–π stacking, which can be explored for material production (Ray and Das, 2015; Chen et al., 2018). More recently, Umerani and co-workers further elucidated the molecular self-assembly of the truncated reflectin variant RfA1TV (63 amino acids) using computational and experimental approaches (Umerani et al., 2020). The variant RfA1TV contains the first linker and first repeating domain of reflectin A1 (RfA1) from *D. pealeii* (Figure 1B). The RfA1TV was soluble in aqueous conditions and monomeric in acidic buffers, conditions that were used to propose the dynamic structural features of ^13^C- and ^15^N-labeled RfA1TV by solution-phase NMR spectroscopy (Figure 1B). The derived model indicated the predominance of random coils and a single α-helix. Upon mechanical stress, RfA1TV self-assembled into different nanostructures with an increased β-sheet fingerprint (Umerani et al., 2020). The same group studied the micro-to-molecular state organization of Ref(2C)4 dissolved in hexafluoro-2-propanol (HFIP) (Xu et al., 2021). Ref(2C)4 is an engineered (113 amino acids) sequence derived from *E. scolopes* R1b with four conserved repeats of Domain 2C, originally described by Dennis et al. (2017). Protein assembly was studied not only in the solution but also in films resulting from protein spin-casting onto substrates. The combination of solution and solid-phase structural characterization methods revealed uniform α-helical regions—associated to hydrophobic residues Tyr, Gly, and Pro—and mobile arrangements of α-helical and β-sheet—associated to positive and negatively charged residues (Arg and Asp) (Xu et al., 2021).

Together, the current structural information for reflectin (poly) peptides is greatly influenced by the various sequences used, as well as by the conditions and additives employed in solubilization, which either hinder or force certain structural arrangements. Recently, we published a paper that shows the potential of the protopeptide sequence to produce sub-microfibers through solution blow spinning in aqueous media. We postulated that this is due to its self-assembly properties; however, the mechanism of the hierarchical assembly remains to be elucidated (Dias et al., 2022). Thus, we set ourselves the challenge of understanding the self-assembly of the most basic reflectin unit—the protopeptide—in the absence of additives, mimicking the aqueous and physiological environment, as well as the protonation effect expected to take place in nature (Morse and Taxon, 2020) (Figure 1C). Protopeptide assembly was studied at distinct hierarchical levels, ranging from the macroscopic scale through the formation of self-sustained hydrogels, to the micro- and nano-scales, and finally to the molecular level (Figure 1D). The supramolecular structures obtained under different conditions were studied, namely, at distinct protonation states, in the presence of aromatic compounds (imidazole) and with mechanical and kinetic triggers. We successfully obtained hydrogels and observed the presence of nanofibers in all conditions. In addition, we verified that thermal stimulus was enough to produce gels independently of pH change and imidazole presence. Furthermore, gel structural characterization and peptide *in silico* simulations contributed to propose a peptide self-assembly model based on β-sheet aggregation driven by interactions mediated through Tyr residues. Considering that the protopeptide is a conserved sequence in the reflectin family, our conclusions contribute to a better understanding of reflectin assembly in physiological conditions. Finally, we also show that protopeptide films are optically active, generating surfaces with diffuse reflectance, similar to that observed in leucophores.

## 2 Materials and methods

### 2.1 Materials

Trizma (Tris base), tricine, sodium dodecyl sulfate, sodium phosphate dibasic heptahydrate, sodium phosphate monobasic monohydrate, sodium acetate, acetic acid, and imidazole were obtained from Sigma-Aldrich. Sodium chloride and sodium hydroxide were obtained from PanReac, hydrochloric acid and 99% ethanol from VWR, and 98% curcumin was obtained from Acros Organics. Acrylamide solutions were purchased from Bio-Rad, and ammonium persulfate (APS) and “N-N-N′-N′-” tetramethylethylenediamine (TEMED) were acquired from NZYTech. The protopeptide (YMDMSGYQ sequence) was chemically synthesized using free terminals and TFA-free at 97% purity by GeneCust (France). Mica used for AFM assays—G250-2 Mica sheets (Cat# AGG250-3, Agar Scientific).

### 2.2 Methods

#### 2.2.1 Peptide self-assembly studies

First, we reproduced the data from Guan et al. (2017) to use these results as a control. Thus, we produced peptide solutions: 3.6 mg/mL and 36 mg/mL in 20 mM Tris-HCl, 150 mM NaCl, and 300 mM imidazole with 0.05% SDS with pH 8. In both cases, the peptide was solubilized immediately. We incubated 3.6 mg/mL for a week at room temperature as described by Guan *et al,* and the 36 mg/mL was incubated until a gel was observed by the glass inversion test in the same conditions. The solution was deposited in mica, and the hydrogel was diluted as described for morphological characterization (see Supplementary Materials—atomic force microscopy).

Second, different strategies were considered to induce self-assembly and formation of hydrogels. To study the effect of hydrophobic and electrostatic interactions in the peptide assembly, we considered four buffers listed in Table 1. To estimate the critical gel concentration, several solutions of peptides at different concentrations were prepared: 2, 3, 5, 10, 16, 26, and 36 mg/mL, in buffers A and B to a final volume of 200 μL. After preparation, the peptide solutions were incubated at room temperature. These solutions were monitored (at intervals of 1 h) by the vial inversion method until gel was formed. To study the kinetic effect in the peptide assembly, 36 mg/mL peptide solutions were prepared in buffers C and D in duplicate to a final volume of 200 μL. After adding the buffer, a white suspension was formed. Tubes were incubated at 90°C for 10 min for complete dissolution, followed by three different strategies: one set of tubes was incubated in ice for 5 min (ICE) and another was sonicated for 5 min (SONIC); after that, both sets were incubated in ice for 5 min; the last set was just incubated at room temperature (RT). These solutions were monitored (in intervals of 1 h) by the vial inversion method until gel formation.

**TABLE 1:** Protopeptide hydrogels produced. The peptide concentration was constant (36 mg/mL).

Gel code	Buffer	Dissolution and gelation condition
**Gel7.5_Im**	Buffer A: 20 mM Tris-HCl and 100 mM NaCl at pH 7.5 with 300 mM imidazole	Peptide dissolved at ambient conditions; hydrogel formed after 5 h
**Gel4_Im**	Buffer B: 20 mM sodium acetate–acetic acid and 100 mM NaCl at pH 4.0 with 300 mM imidazole	
**Gel7.5**	Buffer C: 20 mM Tris-HCl and 100 mM NaCl at pH 7.5	Peptide dissolved under stirring for 10 min at 90°C; hydrogel formed upon cooling down to room temperature after 5 h
**Gel4**	Buffer D: 20 mM sodium acetate–acetic acid and 100 mM NaCl at pH 4.0	

#### 2.2.2 Peptide gel characterization studies

Peptide gels were characterized by various methods, and the details on equipment and sample preparation for morphological (atomic force microscopy and scanning electron microscopy), mechanical, and structural characterization (Rheology, attenuated total reflectance–—Fourier transform infrared spectroscopy (ATR-FTIR), circular dichroism (CD), small angle X-ray scattering (SAXS), and X-ray scattering) are described in detail in Supplementary Materials. Gel4 was further characterized as follows: for evidence on β-sheet formation by the curcumin inhibition test (Yang et al., 2005); to determine the pI of the peptide aggregates by Isoelectric focusing (IEF); and for analysis of the peptide modification after self-assembly by mass spectrometry. These methods are described in Supplementary Materials.

#### 2.2.3 *In silico* studies

Coarse-grained simulations and all-atom molecular simulations were performed to understand the formation of ordered peptide nanostructures at the nano- and molecular levels. Coarse-grained molecular dynamics (CG-MD) simulations were carried out using GROMACS 2019.1 (Lindahl et al., 2019) with the MARTINI v2.2 force field (Marrink et al., 2007). The protopeptide structure was built according to its sequence (YMDMSGYQ) at an atomistic level using PyMOL 2.4.1(DeLano, 2002). The structure was then converted to a coarse-grained one applying the martinize. py script (Martinize, n.d.), comprising the specified parameters for all amino acids (Monticelli et al., 2008). The default peptide charge is −1, corresponding to pH 7.5, and to simulate the pH 4 environment, the generated itp file was edited to match the neutral charge of the peptide at this pH. The backbone beads of N-terminus tyrosine and C-terminus glutamine are changed from Qd to Nda and from Qa to Nda, respectively. Additionally, the aspartic acid side chain bead is changed from Qa to P3, resulting in a neutral overall charge. The peptide structure was minimized in vacuum for 500 steps or until reaching a maximum force of 200 kJ mol^-1^. The 130 molecules of the protopeptide were randomly placed in a cubic box of 12.5 × 12.5 × 12.5 nm. The system was solvated with the force field standard water model and then neutralized by adding Na^+^ ions when needed. For the systems at both pH values, energy minimization for 5,000 steps using the steepest descent integrator was performed. The system was then simulated for 2 μs using a 25 fs timestep (Marrink et al., 2007), with a Berendsen thermostat and barostat (Berendsen et al., 1984) at 298 K and 1 bar, respectively. Systems at both used pH values were also simulated at 363 K. Visual Molecular Dynamics (VMD) program (Humphrey et al., 1996) was used to visualize, and GROMACS (Lindahl and Spoel, 2020) tools were used to analyze structures and trajectories. The aggregation propensity (AP) of the protopeptide was determined after the calculation of the solvent accessible surface area (SASA), using the dedicated GROMACS tool, of the first and last frame of the coarse-grain simulation using the following formula: AP = SASA_init_/SASA_end_.

The prediction of the protopeptide sequence to form fibrils, at an atomistic level, was performed with the 3D profile method from ZipperDB (Goldschmidt et al., 2010). A set of four repetitions of the protopeptide sequence were submitted in the database website. The database provided several outputs, and the top three results with higher aggregation propensity—energy value is equal or below the cut-off of value −23 kcal/mol—were selected to perform atomistic MD simulations of the bundles. To better represent the putative protopeptide fibril assembly, the structures obtained from ZipperDB were edited to insert the corresponding missing residues, resulting in one mono-fibril model and two bi-fibril models. The three obtained models were then submitted to a molecular dynamics simulation protocol. For the all-atom molecular dynamics (AA-MD), simulations of the protopeptide bundle structures were performed at pH 7.5 and 298 K using the GROMACS 2020.6 (Van Der Spoel et al., 2005; Lindahl and Spoel, 2020) with the Amber99SB-ILDN force field (Lindorff-Larsen et al., 2010) and TIP3P water model (Jorgensen et al., 1983). The structures were soaked in a water box extending 1.2 nm from the peptide surface in each Cartesian direction and containing the amount of counterions (Na^+^) necessary for system neutrality. The electrostatic energy was calculated using the PME method (Darden et al., 1993; Essmann et al., 1995); cut-off distances for Coulomb and van der Waals interactions were set to 14 Å; and the equations of motion were integrated with a 2 fs timestep. The calculation procedure was as follows: minimization with 2000 steps of steepest descent followed by 1,000 steps of conjugate gradient; equilibration for 300 ps at 298 K temperature with a Nose–Hoover thermostat and 1 atm pressure using a Parrinello–Rahman barostat; and simulation runs of 250 ns performed in triplicates.

To monitor the simulation, potential energy, total energy, temperature, and pressure values were analyzed throughout the equilibration and the simulation time using GROMACS tools. The last 200 ns of each MD simulation obtained were further analyzed calculating their RMSD and RMSF on α-carbons and the side-chain RMSF. Visualization of the structures was performed using VMD version 1.9.3 (Humphrey et al., 1996) and PyMOL 2.4.1 (DeLano, 2002).

Furthermore, model II was mutated in the tyrosine (wild type) residues to Ala, Leu, Gln, or Phe. The four mutants were simulated at pH 7.5 and both 298 K temperatures as described for the wild type model. Visualization and analysis were performed as previously described.

#### 2.2.4 Optical properties of peptide films

Aqueous solutions of protopeptide and a peptide A1H1 were deposited onto glass substrates and used to produce films. Their optical properties were analyzed by reflectance measurements in the UV–visible region of the light spectrum. The films were characterized by SEM. The detailed methods are described in Supplementary Materials.

## 3 Results and discussion

### 3.1 Protopeptide self-assembly into nanofibers and hydrogels

The self-assembly mechanism of reflectins in animals remains unclear. It is postulated that protein monomers self-assemble into nanoparticles of 20–30 nm, guided by hydrophobic residues that promote π–π stacking interactions (Levenson et al., 2017). In addition, charge interactions are relevant for the dynamic self-assembling mechanism of reflectins mediated by histidine residues present in the linker regions (Levenson et al., 2017). The *in vitro* assembly of full-length reflectins was shown to increase in particle size and for higher-structured aggregates after changes in pH (Levenson et al., 2017) by addition of aromatic compounds, such as imidazole (Guan et al., 2017), or by mechanical shear (Umerani et al., 2020). In the case of the protopeptide sequence YMDMSGYQ (Supplementary Figure S1A, B), it self-assembles into ordered particle-like nanostructures in the presence of imidazole and sodium dodecyl sulfate (SDS) after a week of incubation, as shown in Guan et al. (2017). We confirmed these results by AFM, despite the fact that there is no formation of macroscopic structures, such as hydrogels (Supplementary Figure S2B). The presence of the SDS detergent inhibited charge and ionic strength effects, and the formation of nanostructures was attributed to the interaction between the imidazolium ring and the peptide Tyr side-chain (phenyl ring), through π–π stacking interactions (Guan et al., 2017).

Encouraged by these results, we decided to do a comprehensive study of conditions triggering protopeptide self-assembly into macroscopic structures as hydrogels, which could resemble the conditions found in nature. Cephalopod tissues present a tight acid–base control to maintain pH close to 7.4 to avoid acidosis, which is also ideal for their survival in marine water (Hu et al., 2011; Kaplan et al., 2013), while, in their stomach, the digestion occurs in an acidic medium at pH 3–4 (Bastos et al., 2020). The protopeptide has a pI of 4 (determined experimentally, Supplementary Figure S3) and a specific composition of amino acids. On one hand, this peptide has two tyrosine residues close to the sequence extremes. This is known as the “tyrosine corner,” a structural feature that facilitates interaction between Tyr or other residues through π–π stacking and hydrogen bonds (H-bonds), which often stabilizes protein structures and can induce specific protein conformations (Hamill et al., 2000; Nicaise et al., 2003). In addition, Tyr-rich peptides tend to self-assemble into nanoparticles or nanofibers; thus, Tyr-rich peptides are explored to produce hydrogels and films (Lee et al., 2019). On the other hand, the protopeptide sequence has an aspartic acid residue with a side chain pKa at 3.9, and the N and C-terminals are free, thus allowing electrostatic interactions. Consequently, we considered conditions that could promote i) electrostatic interactions by testing hydrogel formation in physiological conditions at pH 7.5 (peptide charge is −1) and pH 4 (peptide charge is 0); ii) hydrophobic interactions, by assembling gels in the presence and absence of imidazole, as a promoter of π–π stacking; iii) kinetic effect, using sonication to promote mechanical shear.

**FIGURE 2:**
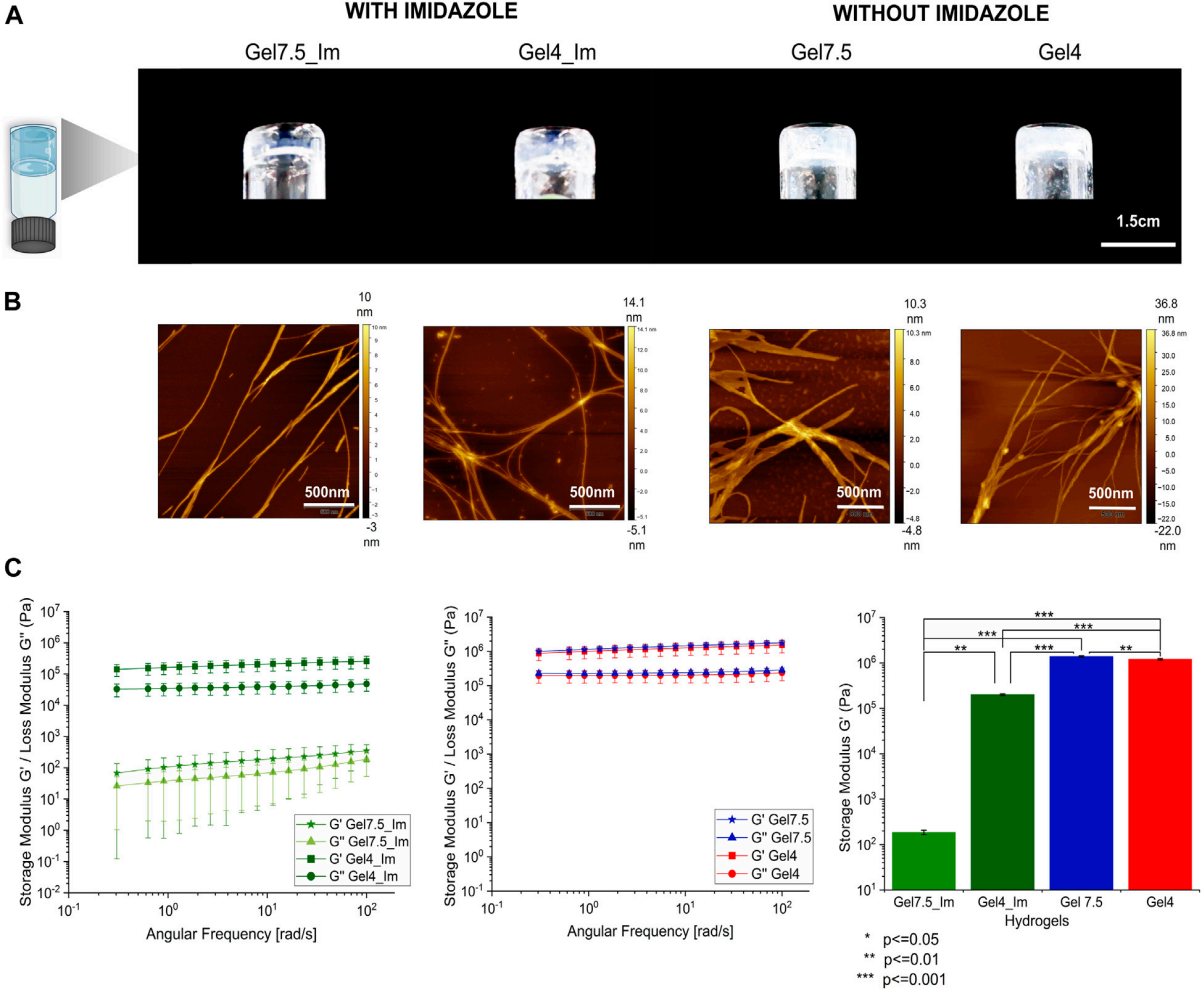
Characterization of macroscopic protopeptide hydrogels. **(A)** Hydrogels at 36 mg/mL were prepared in Gel7.5_Im (buffer A), Gel4_Im (buffer B), Gel7.5 (buffer C), and Gel4 (buffer D). The four hydrogels were characterized by AFM analysis. **(B)** All hydrogels show nanofibers. In addition, by rheology **(C)**, for all gels, the storage modulus (G′) is one order of magnitude higher than the loss modulus (G″) (first graph—gels with imidazole and second graph—gels without imidazole). The mean G′ for each hydrogel is compared between samples (third graph, n = 3 with significance levels and *p*-value below 0.05 as determined by Tukey’s test using Origin 2023 software).

We started by understanding if the detergent SDS was required for the organized self-assembly of the protopeptide into higher hierarchical structures. We determined the critical gel concentration for the protopeptide using buffer A (20 mM Tris-HCl and 100 mM NaCl at pH 7.5 with 300 mM imidazole), mimicking the buffer conditions used by Guan et al. (2017), at pH 7.5 (physiological pH condition in cephalopod tissues and the peptide is negatively charged −1), with imidazole (300 mM) but without SDS addition (Table 1; Supplementary Figure S2A). Under these conditions, the protopeptide was soluble and the gel formation was concentration- and time-dependent, which favor peptide–peptide interactions instead of peptide–solvent interactions. As a reference condition, we selected a protopeptide critical gel concentration of 36 mg/mL at which gel formation took 5 h (Supplementary Figure S2A). This transparent gel (Gel7.5_Im) presented a very soft behavior with variability (storage modulus (G′) of 10^2^ Pa) and was formed by nanofibers with 21.8 ± 8.7 nm in diameter visible in AFM images (Figure 2). At the same protopeptide concentration (36 mg/mL), and as a control experiment, it was also possible to obtain a self-sustained transparent gel with nanofibers observed in AFM (Supplementary Figure S2B), when employing the buffer with SDS, as shown in Guan et al. (2017) (20 mM Tris-HCl, 150 mM NaCl, and 300 mM imidazole with 0.05% SDS at pH 8), but gelation took a week to occur. Thus, we conclude that SDS addition favored peptide solvation, and it was not required for peptide dissolution. SDS reduced the peptide–peptide interactions required for peptide organization into higher-ordered structures, thus explaining the delayed gel formation.

To further understand the effect of the protopeptide charge in hydrogel formation, we compared protopeptide gelation at pH 7.5 and pH 4, for which the peptide is negatively charged (−1) and neutral, respectively. These studies were performed in the presence of imidazole (buffers A and B; Table 1). We determined the critical gel concentration for the peptide in buffer B, which was very similar to that of buffer A (36 mg/mL). We also observed that the protopeptide was soluble and the gel formation was concentration- and time-dependent. For a protopeptide concentration of 36 mg/mL at pH 4, an opaque gel was formed (Gel4_Im), and nanofibers of 17.4 ± 4.4 nm were visible in AFM images (Figure 2 and by SEM in Supplementary Figure S4). These macroscopic structures presented a soft gel behavior, with a G’ of 10^5^ Pa. The low mechanical robustness of Gel7.5_Im compared with that of Gel4_Im might be explained due to charge effects and acid–base pairs used in the buffer composition. In more detail, at pH 7.5, the peptide is negatively charged (−1) and the imidazole (pKa∼6.9, PubChem CID: 795) mainly neutrally charged (Thomason et al., 2015); thus, they can interact through π–π stacking. In contrast, at pH 4, the peptide is neutral, and imidazole is positively charged (+1); thus, there is the possibility of cation–π interactions with Tyr (Rodríguez-Sanz et al., 2015), as well as H-bonds with Tyr, Ser, and Asp. Hence, we conclude that in the absence of charge effects, we could obtain a hydrogel with stronger mechanical properties than the gels formed by the assembly of negatively charged peptide molecules.

Finally, we set out to understand imidazole’s role in the protopeptide assembly process. To achieve this goal, the protopeptide was dissolved in buffers C and D, which are the same as buffers A and B, but without the addition of 300 mM imidazole, at the same peptide concentration (36 mg/mL). The dissolution of the protopeptide in the absence of imidazole was difficult, requiring heating and agitation. After cooling down to room temperature, opaque hydrogels (Gel7.4 and Gel4) with a G’ of 10^6^ Pa were formed. Both hydrogels at pH 7.5 and pH 4 consisted of nanofibers with 31.1 ± 7.1 nm and 23.3 ± 6.2 nm in diameter visible by AFM (Figure 2 and by SEM in Supplementary Figure S4). Therefore, the removal of imidazole allowed the formation of stronger gels at both pH 7.5 and pH 4, suggesting that hydrophobic interactions between protopeptide molecules are critical for the self-assembly of the peptide into nanofibers and gels (macroscopic structures). These gels were observed after a week and after 3 months and were stable without color change (Supplementary Figure S5). Knowing that kinetic effects, as fast heating–cooling treatment and mechanical shear, can further promote fiber assembly, we also tested the protopeptide assembly into hydrogels in buffers C and D, but after fast cooling of the peptide solution or by applying sonication after dissolution. In both conditions, protopeptide hydrogels were formed when peptide solutions were left to cool down at ambient conditions (Supplementary Figure S5C).

Therefore, we concluded that protopeptide molecules can self-assemble as soft gels at 36 mg/mL and under different conditions of additives and pH, with a measured storage modulus (G′) one order of magnitude greater than the loss modulus (G″), characteristic of soft-gels and suggesting viscoelastic behavior. These hydrogels have a G′ in the same range observed for other peptide-based hydrogels reported in the literature (Alakpa et al., 2017; Moreira et al., 2017; Kretschmer et al., 2021; Hamsici et al., 2022). Hydrophobic interactions are important for protopeptide self-assembly, and imidazole acts as a peptide solubilizer while preventing stronger hydrogels to be formed.

### 3.2 Understanding protopeptide self-assembly at the nano- and molecular levels

#### 3.2.1 Experimental models for peptide self-assembly

**FIGURE 3:**
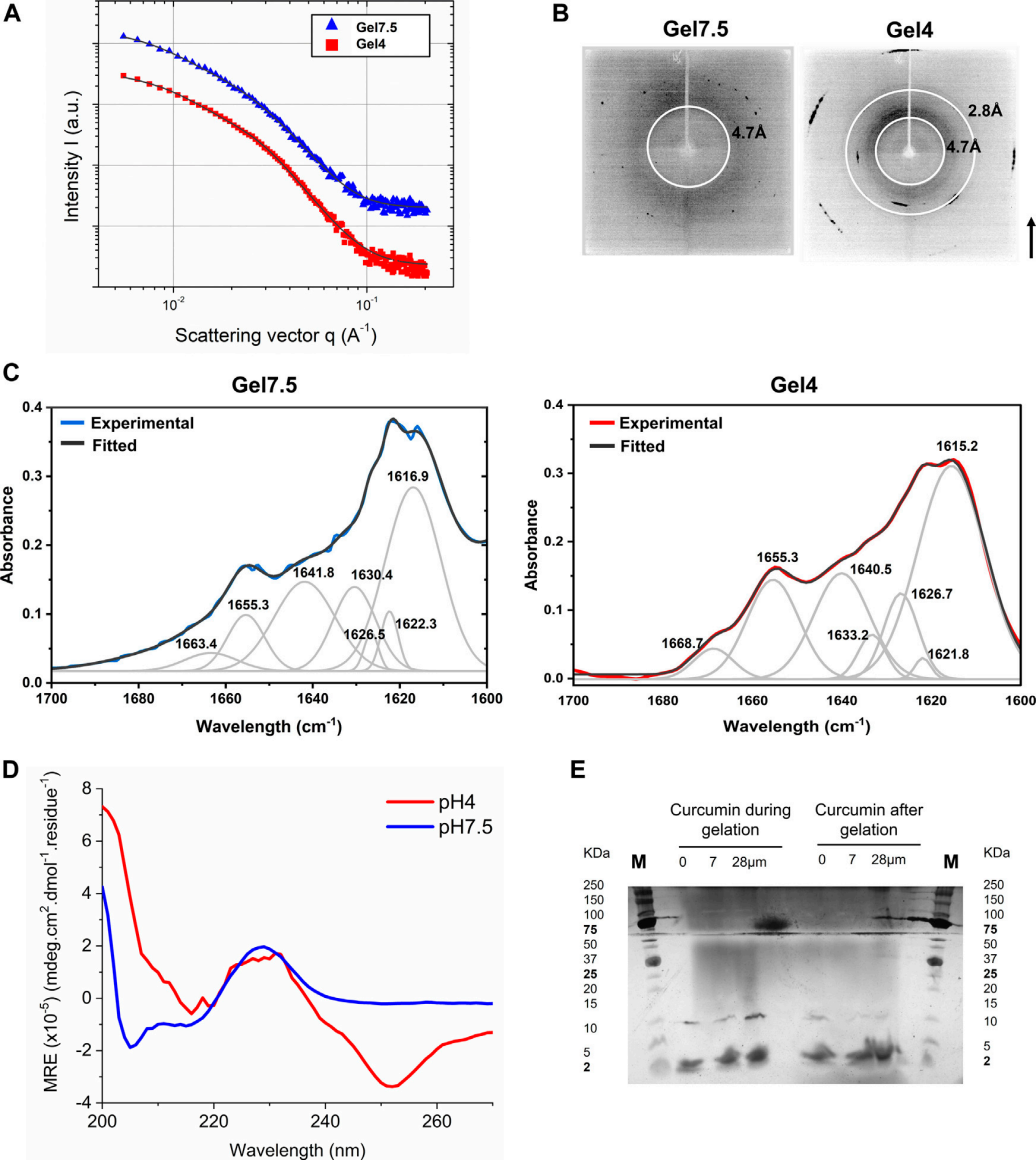
Characterization of hydrogels Gel7.5 and Gel4 without imidazole. **(A)** The small angle X-ray scattering results were fitted to a “gel model”; **(B)** visualization of X-ray scattering in an area detector shows three main scattering rings; **(C)** ATR-FTIR deconvolution results of the Amide I band (1700–1600 nm^-1^) of protopeptide gels produced using different pH conditions (black line: original spectrum; red or blue line: fitted spectra; gray lines: deconvoluted peaks). **(D)** Far-UV circular dichroism spectra of peptide hydrogels (36 mg/mL) at 25°C—Gel7.5 in buffer C and Gel4 in buffer D. **(E)** Native Tris-tricine gel 16.5% with samples from Gel4 incubated with curcumin and without curcumin, showing two main bands 10 kDa and 2 kDa. Marker: Precision Plus Dual Xtra protein ladder (Bio-Rad).

Protopeptide hydrogels formed in the absence of imidazole resulted in mechanically robust materials, possibly indicating a higher-ordered assembly. Consequently, we further studied the protopeptide self-assembly from the nano to the molecular scales for Gel7.5 and Gel4 (Table 1). We started by performing small angle X-ray scattering (SAXS) to determine particle size and shape or agglomeration. After transmission and background subtraction, the radially integrated scattering curves were fitted using a shape-independent model based on the fine-scale polymer distribution within a gel network that involves two characteristic correlation length scales. The short-distance correlation length, a_1_, describes the fluctuations of the chain position to ensure thermodynamic equilibrium. The long-distance correlation length a_2_, known as the mesh size, is due to the polymer being pinned down at the junction points. The fitting results (Figure 3A) suggest that increasing the pH from 4 to 7.5 increases the mesh size a_2_ of the gel from 128 to 231 Å, while a_1_ decreases from 49 to 30 Å (Supplementary Table S1). The a_2_ results agree with the observations from AFM that as the pH increases, the density of fibers decreases (Figure 2B). The a_1_ differences are not substantiated by the rheological properties of the gels, as the G’ value is similar for both gels (Figure 2C).

X-ray scattering rings within resolutions higher than 20 Å were visualized using an area detector (Figure 3B). Overall, signals are stronger in Gel4 than in Gel7.5. Two scattering rings were identified in Gel4, at 4.7 Å and 2.8 Å, whereas only one scattering ring was clearly visible in Gel7.5 (4.7 Å). The scattering rings at 4.7 Å are characteristic of β-sheet-like organization in peptides and proteins (Makin and Serpell, 2005; Cheng et al., 2013; Lampel et al., 2017). The 4.7 Å scattering ring is associated to the distance of alpha carbons between two β-sheets (observed in both Gel4 and Gel7.5), which are stabilized by hydrogen bonds. The scattering ring at 2.8 Å might be associated with spacing between two hydroxyl groups from Tyr, which is a very common and favorable arrangement associated with π–π stacking in protein–protein interactions (Zhao et al., 2015). Taken together, these results suggest that the protopeptide in Gel4 forms more organized and periodic peptide intermolecular interactions, as already suggested by the morphological analysis (Figures 2A,C).

To further understand the structural organization of the peptide molecules, we used ATR-FTIR and circular dichroism. Figure 3C presents the deconvolution of the ATR-FTIR spectra of Gel7.5 and Gel4 in the Amide I region between 1700 and 1600 cm^-1^. In the spectrum of Gel7.5, four bands were observed in the characteristic region assigned to the β-sheet structure at 1617 cm^-1^, 1622 cm^-1^, 1626 cm^-1^, and 1630 cm^-1^. Two bands were assigned to random coil conformation (1642 cm^-1^ and 1655 cm^-1^) and one to β-turn structure (1663 cm^-1^). The ATR-FTIR spectra of Gel4 are characterized by a strong absorbance at 1615 cm^-1^ and lower-intensity bands at 1621, 1626, and 1630 cm^-1^, both assigned to β-sheet structures. Three additional bands were detected, at 1640 cm^-1^ and 1655 cm^-1^, indicative of random coil conformation, and at 1668 cm^-1^, indicative of β-turn, respectively. Overall, peptide assembles into structures with a high content of β-sheets (57% for both Gel7.5 and Gel4), random coil conformation (28% and 39% for Gel7.5 and Gel4, respectively), and a low number of β-turn structures (15% and 3.7% for Gel7.5 and Gel4, respectively) (Supplementary Table S2).

Protopeptide conformation was also analyzed by circular dichroism at low peptide concentrations (0.2 mg/mL, in which peptide molecules are monomeric) and at high peptide concentrations (36 mg/mL, in which the peptide is already assembled as a gel). At low peptide concentrations, we observed a major peak with positive ellipticity at 225–230 nm; this is characteristic of stacking of aromatic amino acids (Miles et al., 2021) and in reflectins is associated with β-sheets structures (Phan et al., 2013; Levenson et al., 2019; Umerani et al., 2020) (Supplementary Figure S6A). The reversibility of the peptide conformation was tested. Supplementary Figure S6B shows that the peptide signal decreased in intensity at 90°C, but increased again after a cooling cycle. This indicates that the stacking of aromatic amino acids in the peptide is reversible with temperature. We further verified that for higher peptide concentrations—Gels7.5 and Gel4—similar spectra are observed but with more features than for low peptide concentrations, indicating a more organized hierarchical structure (Figure 3D). Again, we observed a negative ellipticity peak at 255 nm for Gel4 and for both gels, positive ellipticity peak at 230 nm, a shoulder at 215 nm, a minimum at 205 nm, and an intense peak at 200 nm. These peaks are associated to stacking of aromatic amino acids as Tyr (Miles et al., 2021) (255 nm—close proximity between aromatic side-chain residues in protein tertiary structure; and 230 nm stacking of aromatic residues in proteins secondary structure); n–π* transition peptide backbone (215 nm) (Miles et al., 2021); and finally, π–π* transitions that are frequent in helix conformations (200 nm) (Miles et al., 2021). In full reflectin sequences, similar patterns are usually observed in the CD spectra—a weak positive shoulder at ∼230 nm and a negative peak at ∼200 nm—which are associated to a mixture of β-sheet arrangements and disordered regions (Phan et al., 2013; Levenson et al., 2019; Umerani et al., 2020). Thus, we conclude that the peptide gels have an intense β-sheet content, due to interactions between aromatic residues (e.g., Tyr). In addition, both gels have some helix conformations, in agreement with the ATR-FTIR data.

To further show the presence of β-sheet organized peptide assemblies and characterize their size, we employed the curcumin test. Curcumin is known to inhibit and dissolve protein amyloid-assemblies, as curcumin specifically recognizes peptide sequences assembled in β-sheet secondary structures (Yang et al., 2005). As we show, protopeptide self-assembles into fibers with a high β-sheet content, as observed in AFM, CD, and ATR-FTIR; thus, this is an ideal condition to test the effect of curcumin. It is known that the fibers are composed of fibrils that result from the assembly of several filaments composed of multiple peptide aggregates (Makin and Serpell, 2005). The curcumin test can help in the characterization of these peptide aggregates. Gelation solutions and gels (for Gel4 conditions) were incubated with curcumin (7 and 28 µM) and without curcumin. The solutions gelled within 5 h, and the gels became liquid after curcumin incubation (Supplementary Figure S7), due to the adsorption of curcumin in the gel and possible disassembly of the peptide fibers into peptide aggregates with different sizes (Yang et al., 2005). Figure 3E shows a native Tris-tricine gel with aliquots of these experiments. Two bands are observed. The 10 kDa band is intense in samples without curcumin, and it is present in samples with curcumin during gelation. The intensity of the 10 KDa band decreases in the gel incubated with higher concentrations of curcumin, and a 2 kDa band becomes stronger. The 10 kDa band likely corresponds to an oligomer form of the protopeptide with ∼10 molecules and the 2 kDa band to a dimeric form with ∼2 molecules. These results agree with the observation by Guan et al., (2017), in which the protopeptide formed aggregates of 10 kDa (determined by size exclusion chromatography). We also subjected Gel4 samples to isoelectric focusing in native conditions to assess the pI of the monomeric format and the aggregates (Supplementary Figure S8). The Tris-tricine gel revealed two main bands—a band below 2 KDa with an estimated pI of 4 and a band between 5 and 10 KDa with an estimated pI between 9 and 10. The first band was associated to the peptide in the monomeric format and agrees with the pI determined experimentally (Supplementary Figure S3). The second band is likely related to the multimeric peptide aggregate with exposed side chain residues with a pKa of 9–10, probably tyrosine residues in the N-terminal and on the side chain (pKa 9.11 and pKa = 10.07, respectively). Finally, Gel4 was analyzed by mass spectrometry (Maldi-TOF–TOF), and one major peak was observed m/z 994.36 Da, similar to the monoisotopic mass calculated using a software (993.35 Da, MarvinSketch software) (Supplementary Figure S9). These results indicate that there is no modification in the peptide chain (e.g., methionine oxidation) due to the buffer conditions used.

Overall, our results indicate that the protopeptide assembles in 10 kDa structures with a high contribution of β-sheet arrangements.

#### 3.2.2 Peptide self-assembly model by *in silico* studies

The protopeptide assembles into soft-gels at both pH 7.5 and pH 4, arranged in nanofibers in which the peptide has a predominant conformation in β-sheets. We postulate that the Tyr residues contribute to the formation of intermolecular interactions through π–π stacking and H-bonds. We performed coarse-grained molecular dynamics (CG-MD) simulations to assess the overall peptide organization at the nanoscale and all-atom molecular dynamics (AA-MD) simulations to evaluate the putative organization of several peptides at an atomistic scale.

**FIGURE 4:**
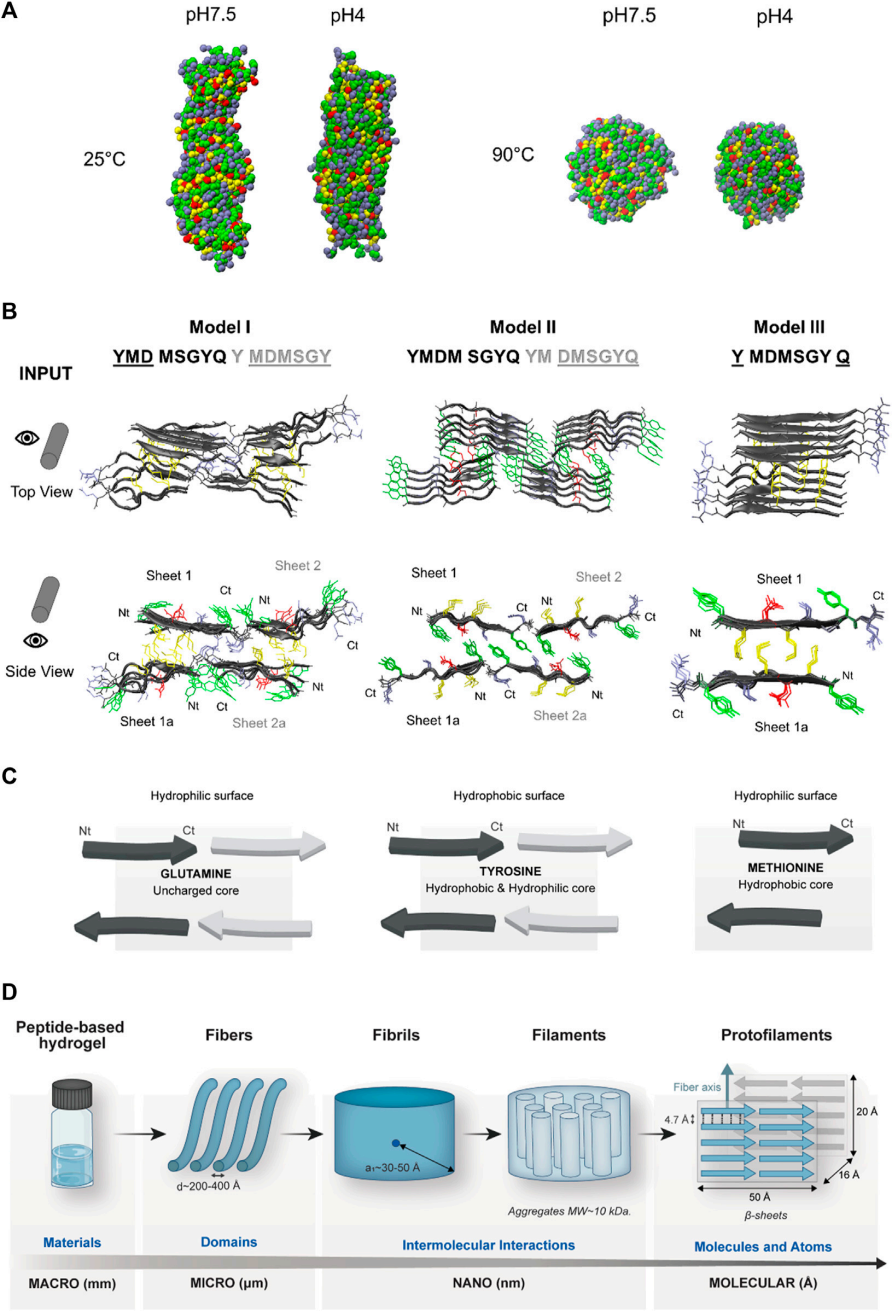
*In silico* studies with the protopeptide and hierarchical assembly models. The amino acid side-chains of the peptide are showed in different colors—Tyr (green), Met (yellow), Asp (red), and Gln, Gly, and Ser (blue). **(A)** Coarse-grained molecular dynamics simulations of 130 molecules of the peptide in a water box in pH 4 (neutral charge) and pH 7.5 (negatively charged) at 25°C and 90°C. At both pHs, at 25°C, there is formation of fibers, and at 90°C, there is formation of aggregates. **(B)** All-atom molecular dynamics simulations: input structures at the top and front view, where different peptide sheets are identified. In bold are identified the amino acids with the highest propensity for aggregation by using ZipperDB (Goldschmidt et al., 2010), in black and gray are the amino acids from two different peptide sheets, and the amino acids with lower aggregation propensity are underlined. **(C)** Schematics of models I, II, and III and the possible properties of each interface region. Images designed in VMD (Humphrey et al., 1996) and PyMOL (DeLano, 2002) visualization software. **(D)** Protopeptide hierarchical assembly model. At the macroscale, peptide hydrogels were studied by rheology. At the microscale, the hydrogels were analyzed by AFM and SEM, revealing nanofibers with the diameter of 200–400 Å. At the nanoscale, SAXS revealed that within these fibers, there were repeated units with a short correlation length between 30 and 50 Å. Furthermore, it was determined in a native protein gel that the peptide forms aggregates with 10 kDa. At the molecular level, the X-ray scattering revealed diffraction characteristics of β-sheet organization, which was further complemented by *in silico* studies. The coarse-grain simulations confirmed the aggregation propensity of the peptide into fibers, and the atomistic level simulations allowed creating a putative model for the aggregation pattern of the protopeptide.

In CG-MD simulations, the peptide assembly was studied at pH 7.5 and 4 at 25°C and 90°C, respectively. At 25°C, the peptide forms fibers with cross-section diameters of 47–50 Å, and at 90°C, we obtained particles with a cross-section diameter of 67–80 Å. This correlates with the experimental observation in which the peptide and hydrogels are dissolved upon heating to 90°C (Figure 4A). The peptide also presented aggregation propensity (AP) values of 5.9 and 7.0 for pH 7.5 and pH 4, respectively (Supplementary Table S3). The AP values are higher at pH 4, which is compatible with the findings of X-ray scattering experiments, where the mesh size has a higher value at pH 4, and the scattering rings are well-defined.

Regarding the atomistic simulations, to analyze the peptide aggregation into β-sheets hinted from experimental data (e.g., CD and ATR-FTIR), beta-amyloid-based models of peptide assembly were created. Protopeptide sequences were submitted to the prediction tool of ZipperDB (Goldschmidt et al., 2010), a database that predicts fibril-forming fragments threading the analyzed sequence into the fibril-forming peptide NNQQNY from the Sup35 prion protein of *Saccharomyces cerevisiae*. When the energy value is equal or below the cut-off of −23 kcal/mol, the analyzed sequence has a high propensity to aggregate into amyloid-like fibrils (Goldschmidt et al., 2010). The models are further analyzed for their propensity for β-sheet secondary structure, shape complementarity (0–1, higher values preferred), and contact area between the two β-sheets. In all resulting models, the amino acids 2 to 5 of the peptide sequence have the highest scores for aggregation and propensity to form amyloid fibrils (Supplementary Figure S10).

The three best ranked aggregation models (Supplementary Table S4) were selected for AA-MD simulation studies. As shown in Figure 4B, these models (I to III) have in total 10 peptide molecules and three different amino acid cores between the two β-sheets: I) uncharged core, glutamine that can promote H-bond interactions; II) hydrophobic and hydrophilic core, tyrosine amino acids that can promote hydrophobic interactions through π–π stacking and H-bond; and III) hydrophobic core, methionine amino acids can form hydrophobic interactions (Figure 4C). After 250 ns of simulation at 25°C, all models demonstrated stable peptide aggregates, except for model I. For models II and III, the stability of the bundles is similar at this timescale with an RMSD of ∼1 and ∼0.7 nm, respectively, and the orientation of the β-sheets is parallel (Supplementary Figure S11). These results suggest that models II and III are the most likely to occur and that aromatic and H-bond interactions should be the key for the assembly of the protopeptide in β-amyloid-like aggregates. The distances collected from X-ray powder scattering data were also observed in the aggregate structures during the simulations, especially in model II. Particularly, the 4.7 Å ring was attributed to the inter-strand distance, which is present in these models. In addition, in models II and III, the distance between two parallel Tyr rings in consecutive peptide chains was estimated in 4.8 Å and can be related with π–π stacking (Zhao et al., 2015) (Supplementary Figure S12). The 2.8 Å ring attributed to the distance between the hydroxyl groups of Tyr was not confirmed in these models, but a pattern of 2.7 Å and 3 Å associated to a distance between hydroxyl groups of Tyr and Ser as well as Tyr and Asp is observed in model II (Supplementary Figure S12). To further comprehend the contribution of Tyr residues for the assembly, four mutant sequences were generated: the Ala mutant in which Tyr was replaced by Ala residues, the Leu mutant in which Tyr was replaced by Leu residues (hydrophobic, aliphatic side-chain), the Gln mutant in which Tyr was replaced by Gln residues (hydrophilic, polar side-chain), and finally the Phe mutant in which Tyr was replaced by Phe residues (hydrophobic amino acid, aromatic side-chain). The assembly models were built starting from model II of the wild-type protopeptide, mutating the selected amino acid residues. These models were used as input for the molecular dynamics simulation for 250 ns at 25°C. We observed the disassembly of the models for all mutants (see input and output structures, Supplementary Figure S13A). The RSMD values over the course of simulation were higher for the mutants (RMSD above 10) when compared with the wild-type sequence (RSMD ∼1) (Supplementary Figure S13B and Supplementary Table S5). The most stable mutants are the Leu and Phe mutants (RSMD between 5 and 10); thus, the hydrophobic core is important for the stability of the self-assembled structures, despite not being the only contributing factor. We also confirmed that the mutant sequences alone do not show propensity for aggregation in ZipperDB software.

Considering these putative models for the assembly of protopeptide and the different mutants, we can conclude that the weakness of soft gels in buffers containing imidazole (Gel7.5_Im and Gel4_Im) is likely due to the presence of imidazole molecules that disrupt intermolecular interactions (π–π stacking and H-bond) between neighboring Tyr amino acids.

Finally, Figure 4D shows the hierarchical assembly model for the protopeptide proposed by the integration of experimental and *in silico* data acquired in this work.

### 3.3 Optical properties of peptide films

Surfaces with fibers are known to produce diffuse reflectance phenomena (Gamage et al., 2021), and nanofibers promote a wider light scattering than nanoparticles, due to a higher aspect ratio and random orientation of the fibers (Chen et al., 2012). We found out that the protopeptide assembles as nanofibers; thus, we hypothesized that protopeptide-derived materials could yield diffusive reflective surfaces.

**FIGURE 5:**
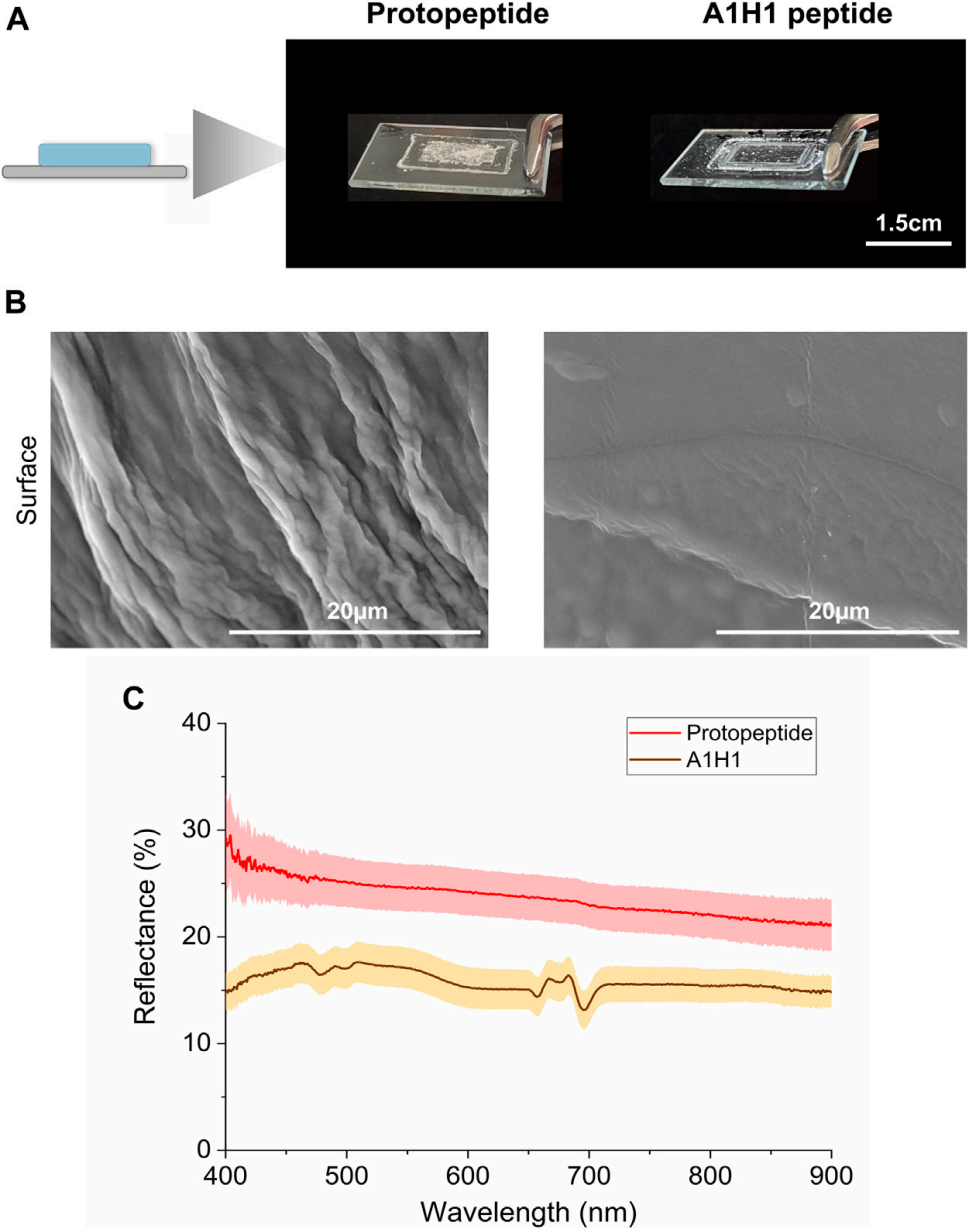
Optical characterization of peptide films. **(A)** Protopeptide and A1H1 peptide films deposited over a glass slide. **(B)** SEM characterization of the protopeptide (left panel) and A1H1 (right panel) films. **(C)** Diffuse reflectance plots of the protopeptide (in buffer D) and A1H1 peptide with the sequence AATAVSHTTHHA (in 95:5 (v/v) water in acetonitrile) films in the visible region of the light spectrum. The lines represent the average signal for each sample (n = 2), and the standard deviation is represented as a shade.

We deposited the peptide solutions over a glass surface and measured the reflectance in the visible spectrum (Figure 5). The optical properties of the films (Figure 5A) were compared with those obtained from a control peptide similar in size (A1H1 peptide—AATAVSHTTHHA, Supplementary Figure S1C, D). The A1H1 peptide derives from suckerins (proteins present in squid ring teeth), and it is well-characterized and known to assemble into β-sheet structures (Hiew et al., 2017; Sun and Ding, 2020). Protopeptide films are translucid with 25%–30% broad-band reflectance, while the A1H1 peptide presented 15% reflectance (transparent films). The difference in the diffusive reflectance signal between the two peptide films (both self-assemble into nanofibers showing β-sheet conformation (Hiew et al., 2017; Sun and Ding, 2020)) can be mostly explained by the fact that protopeptide-based films present fibers at the surface, while the A1H1-based films show smooth surfaces with very thin fibers, as observed by SEM (Figure 5B and Supplementary Figure S14). In addition, the content of polarizable amino acids (25% Tyr and 25% Met) in the protopeptide is higher than that in the A1H1 peptide (25% His), which may also add to explain the differences observed (Zhao et al., 2011; Khago et al., 2018).

## 4 Conclusion

We investigated the assembly of the protopeptide YMDMSGYQ as a basic unit of reflectin proteins. From the different conditions tested in this work, we concluded that the ideal self-assembly condition into self-sustained hydrogels is at a peptide concentration of 36 mg/mL in 20 mM sodium acetate–acetic acid, 100 mM NaCl at pH 4, using a heating and cooling cycle as the trigger, and without any other additives (e.g., SDS or imidazole). After several complementary characterizations from macro-to-molecular scales, it was possible to propose a hierarchical self-assembly model for this peptide (Figure 4D). At the macroscopic scale, rheology shows that these hydrogels have typical viscoelastic properties of peptide-based hydrogels. At the microscopic level, through AFM and SEM, we obtain information that the hydrogel is formed by nanofibers, with an estimated diameter between 200 and 400 Å. At the nanoscale, through SAXS, it was observed that these fibers are composed of smaller peptide units of about 30–50 Å. At the molecular scale, X-ray scattering of dried hydrogels revealed scattering rings characteristic of β-sheets and π–π stacking. Furthermore, the peptide propensity to assemble into fibers was modeled *in silico*. The obtained putative model II presented the most stable aggregation pattern of peptide chains in parallel β-sheets, supporting experimental observations. Thus, we established that the most relevant interactions for peptide self-assembly are hydrophobic (through π–π stacking) and H-bond, mostly mediated by tyrosine residues. Finally, and considering the fibers observed in protopeptide-derived materials, we assessed the optical properties of protopeptide films, revealing a higher diffusive reflectance signal when compared to films produced by a control peptide.

The present work defines protopeptide self-assembly conditions without additives, contributing for a crucial understanding of reflectin assembly mechanisms and design of reflectin-based materials.

## Data Availability

The original contributions presented in the study are included in the article/Supplementary Material; further inquiries can be directed to the corresponding authors.
