# Supplementary material for: Hierarchical self-assembly of a reflectin-derived peptide
Source: Front Chem. 2023 Sep 21;11:1267563. doi: 10.3389/fchem.2023.1267563 (PMC10552760; doi:10.3389/fchem.2023.1267563)
Supplement: Supplementary file 1 [file DataSheet1.docx]

Supplementary Material

**Hierarchical self-assembly of a reflectin-derived peptide**

**Ana Margarida Gonçalves Carvalho Dias^1,2*^, Inês Pimentel Moreira^1,2^, Iana Lychko^1,2^, Cátia Soares^1,2^, Arianna Nurrito^1,2^, Arménio Jorge Moura Barbosa^1,2^, Viviane Lutz-Bueno^3,4^, Raffaele Mezzenga^3^, Ana Luísa Carvalho^1,2^, Ana Sofia Pina^1,2^, Ana Cecília Afonso Roque^1,2 *^**

^1^Associate Laboratory i4HB – Institute for Health and Bioeconomy, School of Science and Technology, Universidade NOVA de Lisboa, 2829-516 Caparica, Portugal

^2^UCIBIO – Applied Molecular Biosciences Unit, Department of Chemistry, School of Science and Technology, Universidade NOVA de Lisboa, 2829-516 Caparica, Portugal

^3^Department of Health Sciences and Technology, ETH Zürich, 8092, Zürich, Switzerland

^4^Paul Scherrer Institute, 5232, PSI Villigen, Switzerland

*** Correspondence:**Corresponding Authors
margarida.dias@fct.unl.pt; cecilia.roque@fct.unl.pt

# Supplementary Methods

**1.1 Peptide Gels characterization**

Morphological characterization - Atomic force microscopy (AFM) analysis was performed using an Asylum Research MFP-3D Stand Alone AFM system. Peptide soft-gels (36 mg/mL) were prepared in the following conditions: Buffer A (Gel7.5_Im), Buffer B (Gel4_Im), Buffer C (Gel7.5) and Buffer D (Gel4). Soft-gels were diluted 1:4 and 1:10 and 10 μL of the resultant dilution were loaded into mica layer and dried overnight in a dust free environment. Same strategy was followed for buffers only solutions. Measurements were performed in tapping (alternate contact) mode in air, using commercially available silicon AFM probes (Olympus AC160TS; k = 26 N/m; f0 = 300 kHz) with a minimum resolution of 256x1024 points. The resulting AFM images were planefitted and analyzed in the Gwyddion software (http://gwyddion.net/). Images analyzed were 10*10 μm up to 2*2 μm size and in Height Retrace mode.

Scanning electron microscopy (SEM) observations were conducted on a Carl Zeiss AURIGA CrossBeam (FIB-SEM) workstation coupled with energy dispersive X-ray spectroscopy (EDS, Oxford X-Max 150 detector with Aztec software). A drop of the hydrogel diluted 1:4 was deposited in a carbon layer and let to dried in a closed petri dish at room temperature. In the next day, samples were coated with Palladium/Gold conductive film to avoid charge effects and analyzed.

Mechanical and Structural characterization - Mechanical properties were studied using an Anthon Paar rheometer MCR 102 with temperature controlled at 20°C. A measuring cone CP25-2, with a 25 mm diameter and 2° angle, was used. Before the oscillatory rheology measurements, a strain at the linear viscoelastic regime was chosen from amplitude sweep measurements, which was 0.001 for all the samples. Then, triplicates of the same sample were carried out by carefully loading the samples on to the rheometer and the average was determined. The data was processed by calculating the mean values and standard deviation in OriginPro2023 software. Furthermore, the G' data was further statistically characterized to determine the statistical variance between hydrogels. For that was applied the pair wise comparison with Tukey test and considered a variance significance below 0.05 (for p-values<0.05, data was considered different). The graphs data was plotted using Paired Comparison plot app available in OriginPro2023 software.

All hydrogels were measured by small angle X-ray scattering (SAXS) using the methodology described in [10,18]. Shortly, SAXS was performed with Rigaku MicroMax-002+ micro-focused beam (4 kW, 45 kV, 0.88 mA). The copper Kα energy with λe = 1.54 Å was collimated by three pinholes onto a beam size of 700 × 700 μm^2^. A two-dimensional argon-filled Triton detector was used for collecting SAXS patterns, over an active range q = 0.001–0.200 Å^−1^. Different soft-gels – Gel7.5_Im, Gel4_Im, Gel7.5 and Gel 4 - were loaded in a capillary and measured for 3h. After transmission and background subtraction, the radially integrated scattering curves were fitted with a shape independent model (gel_fit) with SASView (https://www.sasview.org/docs/user/models/gel_fit.html). The fitting parameters are shown in Table S1.

Hydrogels Gel7.5 and Gel4 were dried under a strong magnetic force field and further analyzed by X-ray scattering. The X-ray scattering images were collected on a microfocus I𝜇S Bruker D8 Venture CuK𝛼 diffractometer operated at 50kV and 1mA and coupled to a Photon 100 CMOS detector.

Soft-gels Gel7.5_Im, Gel4_Im, Gel7.5 and Gel4 were prepared in buffers with deuterium water were analyzed using FTIR. Spectra were recorded using Spectrum Two with UTAR two adapter from Perkin Elmer. Scans were recorded in absorption mode and as a background air was used. In total 25 scans were obtained and averaged with the range from 4000-400 cm^-1^. Data was processed and specific peaks were analyzed, specifically, Amide I signal (1700-1600 cm^-1^) to access the secondary structure of peptide in the soft-gels. All deconvolutions of Amide I bands were carried out using Origin 2021b software with Peak Deconvolution tool (v.1.6). The position and number of peaks were defined from the results of second derivatives spectra with the application of the Quadratic Savitzky-Golay smooth derivative method (2nd polynomial order) and 20-35 points. A Gaussian model was selected for the band shape and the bandwidth. The assignment of peaks to the secondary structure was based on the characteristic position of each secondary structure described previously in the literature [2–4]. The relative secondary structure composition was calculated by dividing the individual deconvoluted peak area by the total Amide I peak area and multiplied by 100 [5].

Protopeptide secondary structure and its thermal stability was studied in different buffer compositions through Circular Dichroism (CD). Peptide was prepared at 0.2 mg/mL in Buffer C, Buffer D and Buffer C and D with 2 mM Imidazole. The spectra were recorded using a Chirascan qCD spectropolarimeter. The full spectra were acquired in a cuvette with a light path of 0.2 mm at different temperatures 37 °C (to ensure peptide solubility), 90 °C and 37 °C after denaturation. In the wavelength scan mode, using 1 nm bandwidth with a step size of 1 nm between 195 to 300 nm. Three accumulations were acquired for each sample, averaged, and smoothed. The resulting data were baseline corrected for solvent contributions. Furthermore, a denaturation curve for peptide in Buffer D was made between 5 to 89 °C. Protopeptide self-assembly within the soft-gels was studied through circular dichroism at 25 °C. Soft-gels Gel7.5 and Gel4 were transferred to a demountable U-shaped cuvette with a lightpath of 2 mm. The spectra were recorded in the same conditions the peptide solutions, except for the temperature conditions, for soft-gels was used 25°C.

Inhibition of β-sheet aggregates with Curcumin - The protocol was adapted from [6] to soft-gel Gel4. A 36 mg/mL peptide solution was prepared in Buffer D in a total volume of 100μL, this solution after heating at 90°C was divided in six vials with 10 μL each. A solution of curcumin was prepared at 98mg/mL in 100% ethanol (266mM). The six vials were divided in two conditions: i) addition of curcumin at 0, 7 and 28 mM from stock and allow soft-gel formation at room temperature; and ii) allow soft-gel formation at room temperature (up to overnight) and then add curcumin at 0, 7 and 28 mM and incubate for 4h at 37 °C. At the end of incubation, Buffer D was added to all solutions, to bring the final volume to 40μL. All vials were centrifuged to 14000 g, 10 min at 4 °C, and the supernatant (~30 μL) of the solutions was transferred to a new vial and mixed with 30 μl Tris-Tricine sample buffer (200mM Tris-HCl pH6.8, 2% SDS, 40% Glycerol, 0.04% commassie blue R-250). Tris-Tricine gels were produced manually and are composed by three parts: 15% acrylamide resolving gel, 10% acrylamide spacer gel and 4% acrylamide stacking gel. 15% acrylamide resolving gels were prepared by mixing 5.33 mL of Solution I (30% acrylamide/bis solution 19:1), 4.3 mL of 2.5 Tris pH 8.8 solution, 0.22 mL of distilled water, 0.006 mL of TEMED and 0.1 mL of 10% APS. To prepare the 10% acrylamide stacking gels the following solutions were mixed: 0.835 mL of Solution II (30% acrylamide/bis solution 29:1), 1.4 mL of 2.5 Tris pH 8.8 solution, 0.225 mL of distilled water, 0.0015 mL of TEMED and 0.0375 mL of 10% APS. 4% acrylamide stacking gels were prepared by mixing 0.33 mL of Solution II, 0.38 mL of 2.5 Tris pH 8.8 solution, 1.71 mL of distilled water, 0.0025 mL of TEMED and 0.075 mL of 10% APS. 20μl of each solution was loaded into a native Tris-Tricine gel 16.5% and used as marker the Dual Xtra protein ladder (Biorad). Gels were run in buffer - 100mM tris, 100mM tricine, 0.1% SDS pH8.3 - at a constant 0.04mA for 2h. The gels were stained with Silver Stain PLUS kit (Biorad) and documented in GelDoc (Biorad).

Isoelectric focusing (IEF) of Gel4 - The method used was as described for 2D electrophoresis kits (Biorad) using native conditions between pH3 and pH10. For electro focusing was used 169μg of Gel4 that were analyzed using ReadyStrip IPG strips pH3-10 7cm (cat#1632000) in a PROTEAN IEF system (Biorad). To ensure native conditions, all buffers were prepared without chaotropic agents, detergents and 1,4-dithiothreitol (DTT). In more detail: Hydration Buffer - 0.2% (v/v) Biolyte 3/10 in water; Equilibration Buffer I and II – Tris-Tricine running gel with 20% (v/v) Glycerol. For electrophoresis was followed the same methods as in curcumin assay. In short it was used a Tris-Tricine gel 16.5% with a Precision Plus Dual Xtra protein ladder (Biorad) and the gel was silver stained.

Mass Spectrometry of Gel4 - The peptide samples were desalted/concentrated using POROS C18 (Empore, 3M) and eluted directly onto the MALDI plate using 1 μl of 5 mg/ml CHCA (alpha-cyano-4-hydroxycinnamic acid, Sigma) in 50 % (v/v) acetonitrile and 5 % (v/v) formic acid. The data was acquired in positive reflector MS mode using a 5800 MALDI-TOF/TOF (AB Sciex) mass spectrometer and using TOF/TOF Series Explorer Software v.4.1.0 (Applied Biosystems). External calibration was performed using CalMix5 (Protea). The raw MS data was analyzed using Data Explorer Software v. 4.11 (ABSciex).

**1.2. Peptide films and characterization of optical and morphological properties**

Reflectance – Protopeptide films’ optical properties were compared with A1H1 peptide, derived from suckerins (marine proteins). The peptide A1H1 (AATAVSHTTHHA) was chemically synthesized with free terminals and TFA free with purity>97 % by Genecust (France).

Films were prepared using 30mg/ml peptide solutions as follows: protopeptide was dissolved in Buffer D (20 mM Sodium acetate-acetic acid, 100 mM NaCl at pH 4.0), protopeptide required heating for dissolution as described for hydrogels preparation; and A1H1 was dissolved in 95:5 (v/v) water in acetonitrile, ideal buffer for dissolution and β-sheet formation. The solutions were deposited (100 µL per layer) in a glass slide within a 3D printed rectangular support with 1 cm x 0.7 cm and a height of 0.3 cm and incubated room temperature until dry (about 2h). In total, it was added 5 layers of peptide solution.

Reflectance spectra were measured in Ultraviolet-Visible (UV-Vis, 300-1000 nm) using a spectrophotometer FLEX-Res+ Vis-NIR (light source: LS-W20 Tungsten) coupled to reflection probe (RP-NIR-400 μm, 2 m) both from Sarspec (Portugal). Optical fiber was positioned with an angle of incidence of 45º. All measurements were recorded in the wavelength range of 300 - 1000 nm, with scans intervals of 1 nm and referenced to a Sarspec standard (fused silica substrate coated with Al-MgF2 for high specular reflectivity). Each sample was measured in duplicate and at four different regions of the films to examine the reproducibility of the conditions and homogeneity among the films. All spectra were collected at room temperature with a relative humidity of 50%.

Morphological characterization – The protopeptide and A1H1-based films fabricated in glass were analyzed by Scanning electron microscopy (SEM). The observations were conducted on a Hitachi TM 3030Plus Tabletop. Samples were coated with Palladium/Gold conductive film to avoid charge effects and analyzed.

# Supplementary Figures and Tables


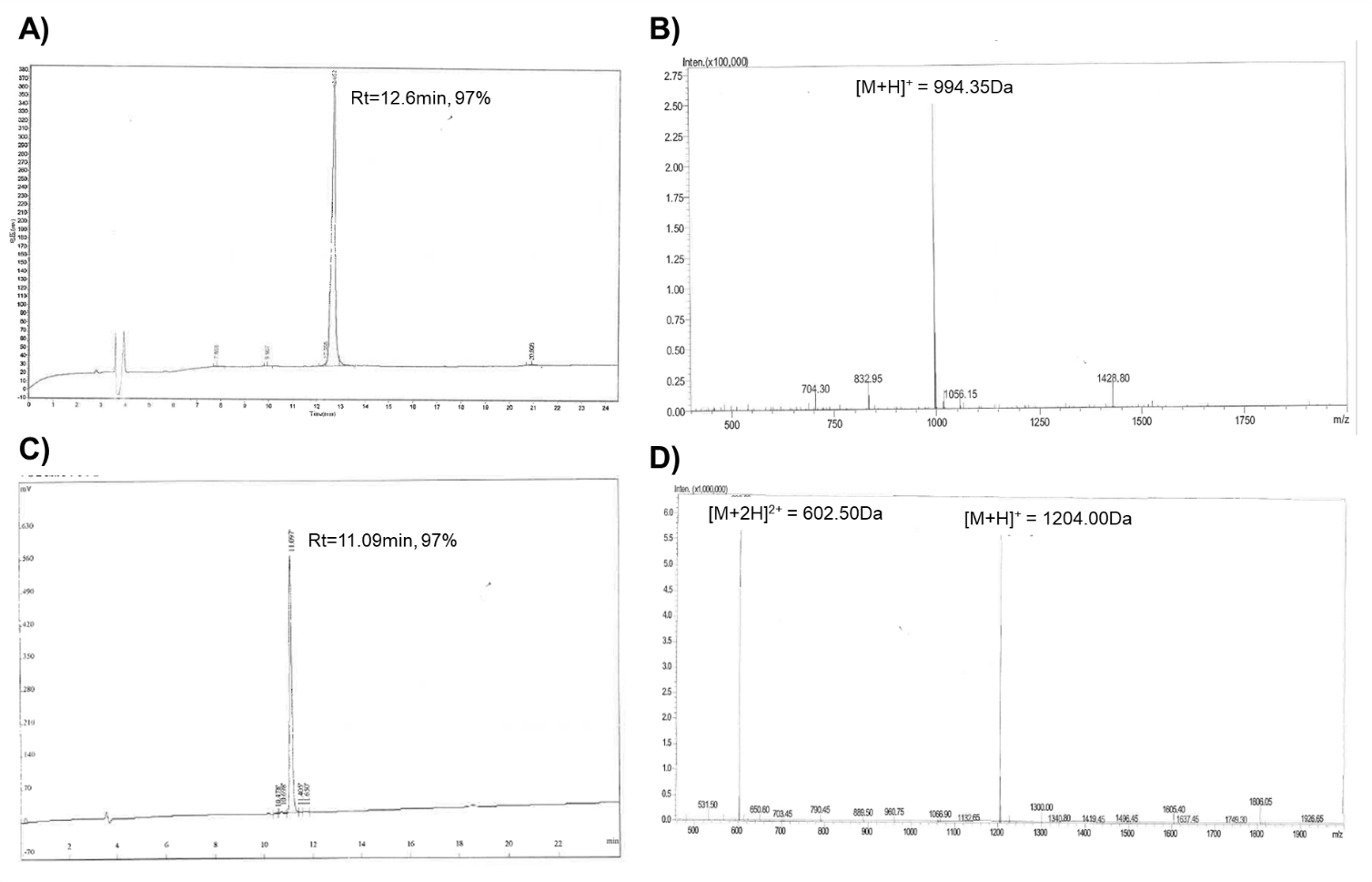


**Figure S1 –** Characterization of peptides used in this work. Protopeptide (YMDMSGYQ). A) HPLC purification shows one major peak identified Rt=12.6 min with 97% intensity. B) Identity was confirmed by ESI-MS with [M+H^+^] = 994.35 Da. A1H1 (AATAVSHTTHHA). C) HPLC purification shows one major peak identified Rt=11.09 min with 97% intensity. D) Identity was confirmed by ESI-MS [M+H^+^] = 1204.00 Da. The data was provided by Genecust upon synthesis of the peptide.

**
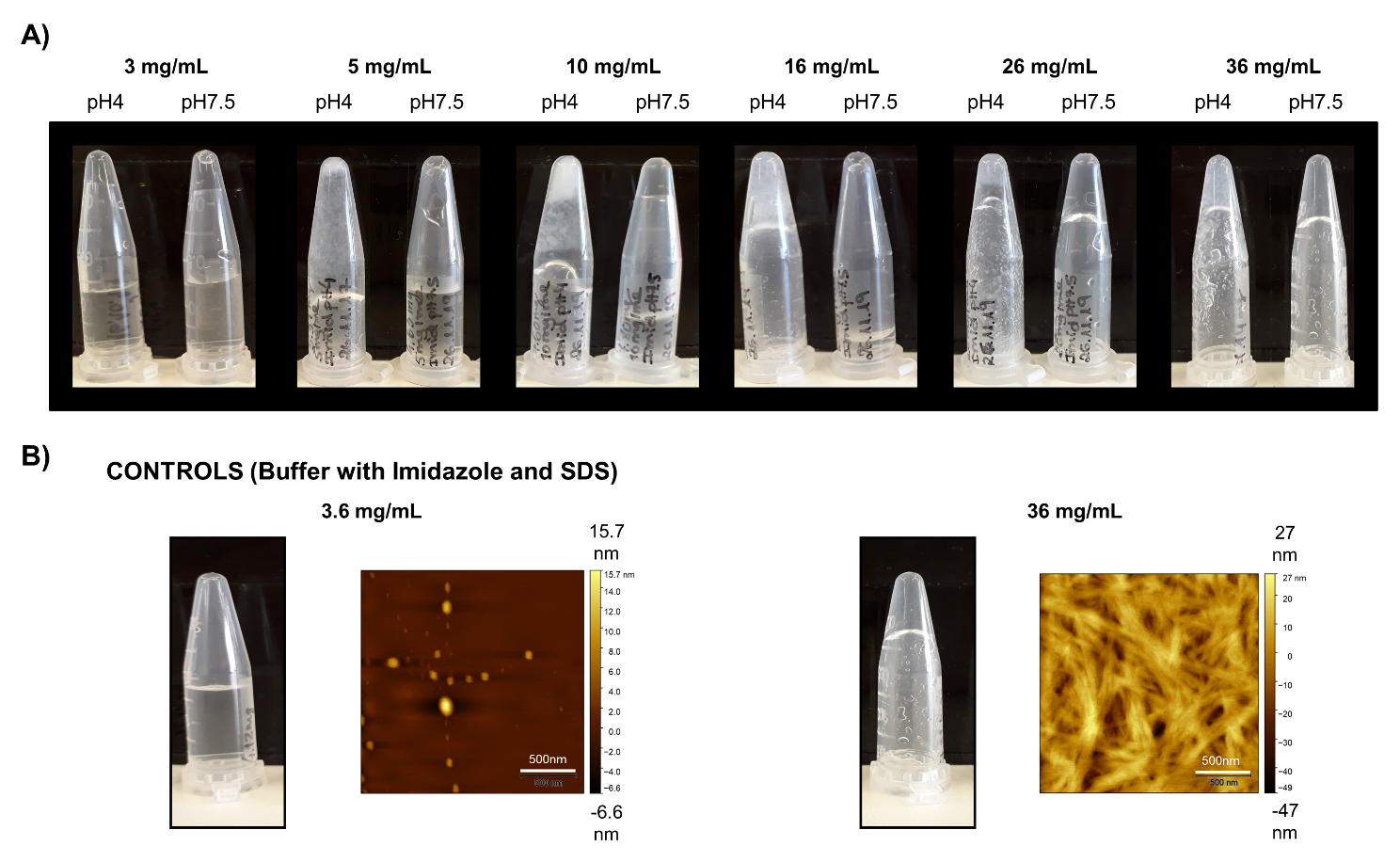
**

**Figure S2** – A) The critical gelation concentration was determined at different peptide concentrations in: pH7.5 (Buffer A, 20mM Tris-HCl, 100mM NaCl at pH7.5 with 300mM Imidazole); and pH4 (Buffer B, 20 mM Sodium acetate-acetic acid, 100 mM NaCl at pH 4.0 with 300 mM Imidazole). The gel formation was monitored by vial inversion method, at 5 hours only 36 mg/mL is a self-sustained gel (at pH4 is an opaque gel and pH7.5 is a transparent gel). B) As controls and to reproduce results found in Guan *et al* (Guan et al., 2017), we prepared peptide solutions at different concentrations (3.6 and 36mg/mL) in buffer with Imidazole and SDS (20mM Tris-HCl, 150mM NaCl, 0.05% SDS pH8, with 300mM Imidazole). The peptide dissolution and gel formation time were monitored by the vial inversion method, 3.6mg/mL is a liquid and 36mg/mL we observe the formation of a gel. These samples were analyzed by Atomic Force Microscopy. For 3.6mg/mL is observed nanoparticles and for 36mg/mL is observed nanofibers.

**
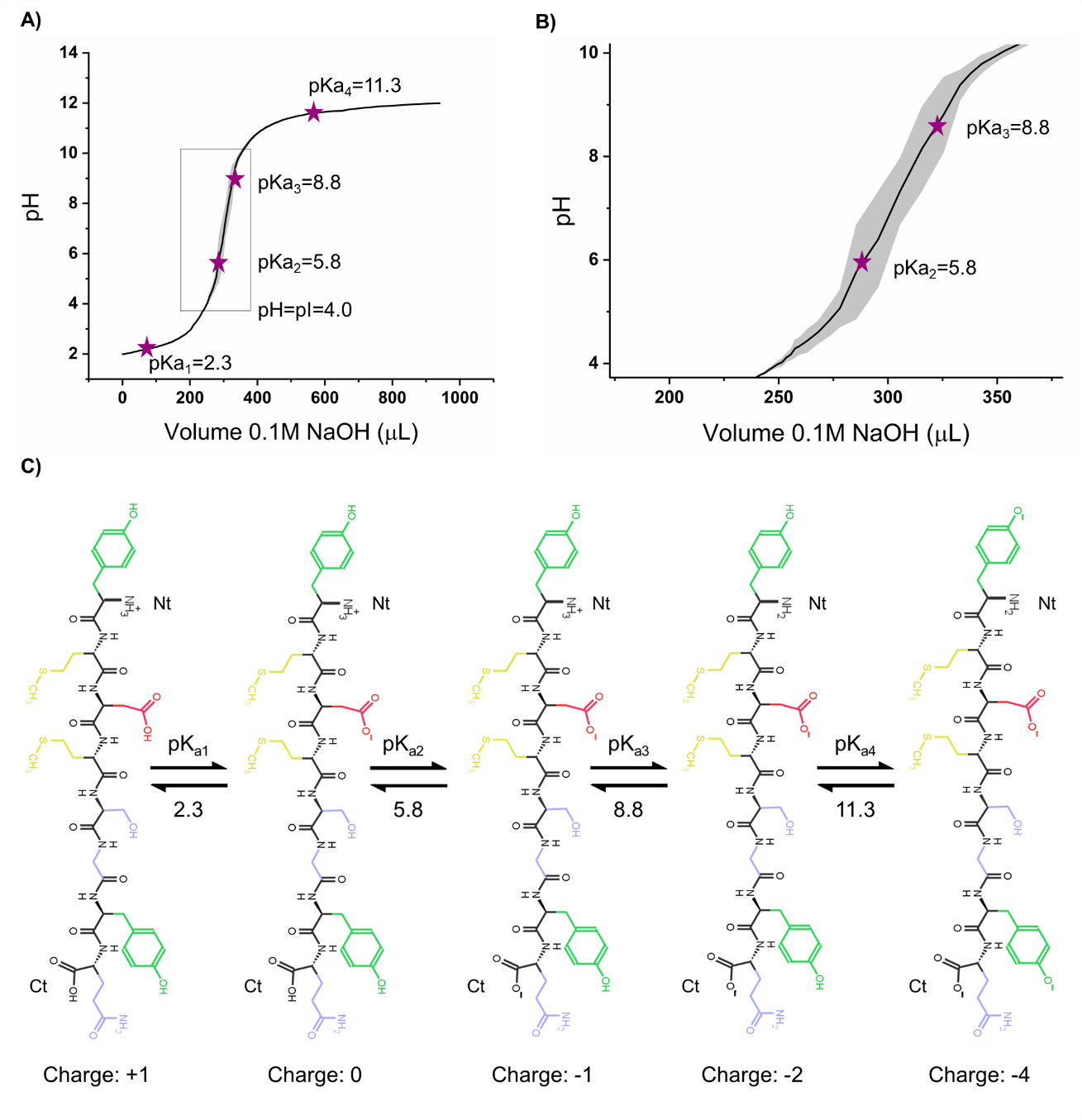
**

**Figure S3** – Peptide titration curve. A) The titration curve was acquired between pH2 and pH12 using a 2mM protopeptide solution in water. The pH was raised with increments of 0.1M NaOH, as described in (Nelson et al., 2021). In the curve were identified different pKa that result from the different charged species in solution; B) Zoom in pH4 to pH10; C) Equation of charged species in solution between pH2 and pH12. The N-terminal (Nt) and C-terminal (Ct) are identified in the structure. Peptide structures were draw and the chemical properties of the peptide were analyzed using Marvin 6.2 software (ChemAxon, <https://www.chemaxon.com)>.


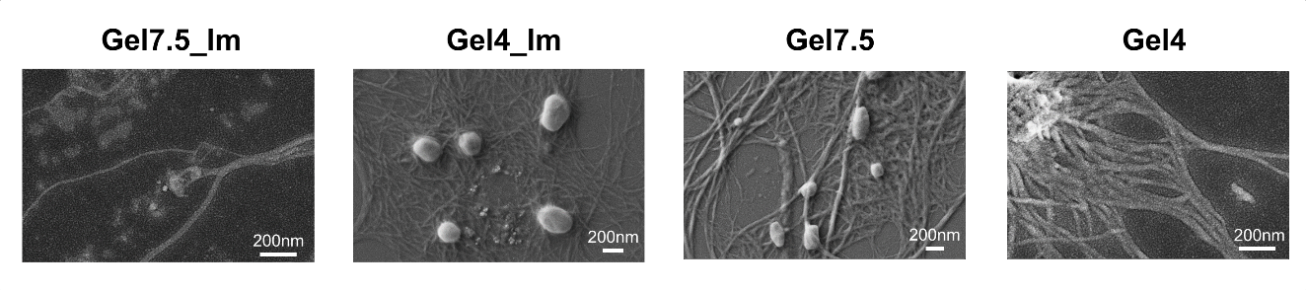


**Figure S4** – SEM analysis of protopeptide hydrogels prepared in different conditions. The presence of nanofibers is visible in all hydrogels.


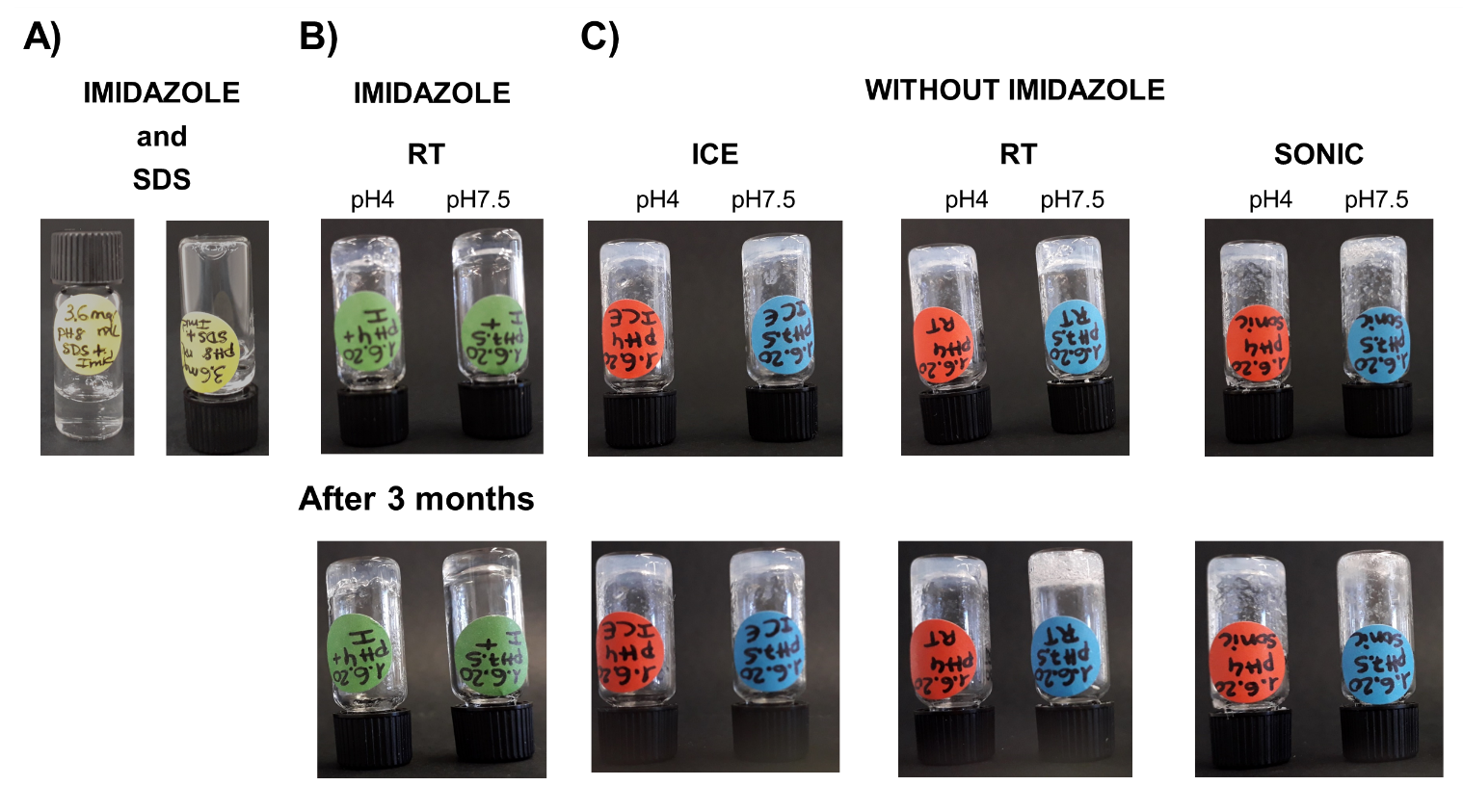


**Figure S5 –** Comparison between different peptide preparations. The gelation time was monitored by the vial inversion method. A) As a control, we use 3.6mg/mL peptide in buffer described by Guan *et al* (20mM Tris-HCl, 150mM NaCl, 0.05% SDS pH8, with 300mM Imidazole). In this condition we always obtain a transparent liquid, and no gel is formed independently of the incubation time. However, after a week, in AFM particles-like nanostructures are visible, as reported by (Guan et al., 2017). In B) and C), we evaluated the stability of the different hydrogels prepared at 36mg/mL overtime. Final evaluation after 3 months incubation at room temperature in the dark. B) Hydrogels prepared in Buffer A and B; C) Hydrogels prepared in Buffer C and D and in different preparation conditions: “heat shock” and mechanical shear. ICE – peptide solutions were heated at 90°C and cooled down on ice; RT – peptide solutions heated and cooled down at room temperature; and SONIC – peptide solutions were heated and sonicated before cooling down in ice.

**Table S1 -** SAXS fitting parameters:

| **Sample Hydrogel** | | **Gel7.5** | **Gel4** | **Units** |
| --- | --- | --- | --- | --- |
| **Parameters** | Guinier | 2.6 | 0.9 | a.u. |
|  | Lorentz | 1.7 | 2.1 | a.u. |
|  | fractal | 4.6 | 4.3 | -- |
|  | a_1_ | 231.25 | 128.3 | Angstrom |
|  | a_2_ | 30.1 | 49.9 | Angstrom |

**Table S2 -** Deconvolution ATR-FTIR data for the hydrogels

| **Secondary Structure Assignment** | | **β-sheet** | **Random-coil** | **β-turn** |
| --- | --- | --- | --- | --- |
| **Sample Hydrogel** | Gel7.5 | 57.0 % | 27.9 % | 15.1 % |
|  | Gel4 | 57.2 % | 39.2% | 3.7 % |

**
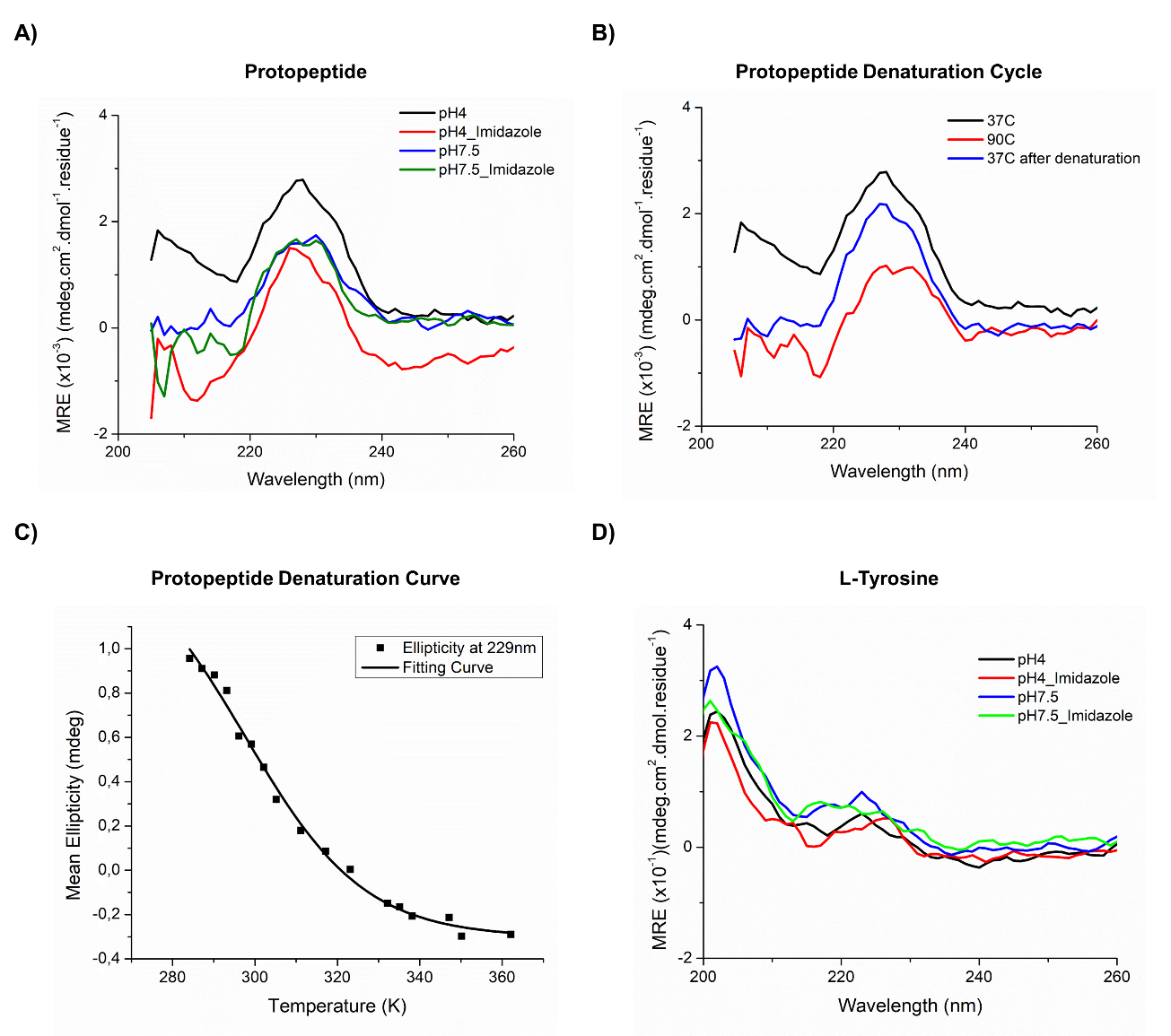
**

**Figure S6** – Circular Dichroism assays with protopeptide. A) Far-UV CD spectra of peptide (0.2mg/mL) at 37°C in different buffers: Buffer C and Buffer D with or without 2mM Imidazole. B) Far-UV CD spectra of peptide (0.2mg/mL) at 37°C in Buffer D before and after denaturation at 90°C, the signal of the peptide is recovered. Only values until 205nm are available due a signal saturation in the equipment.


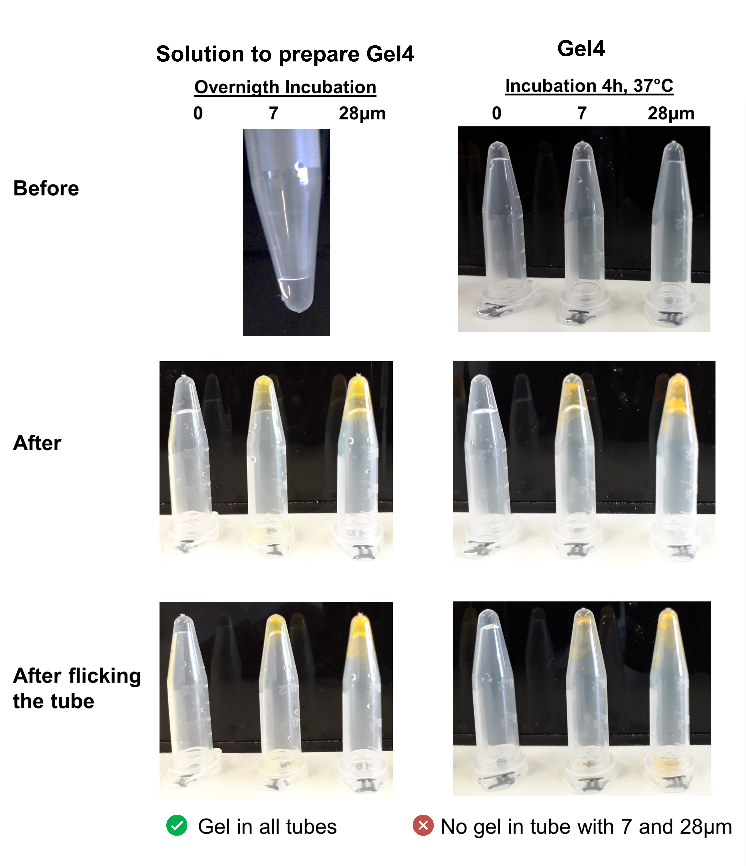


**Figure S7** – Assays with curcumin to inhibit β-sheet secondary structures. A) Gel4 solution before gelation and Gel4 after gelation were incubated with curcumin and without curcumin. The curcumin destroyed the gel after incubation for 4h at 37°C.

**
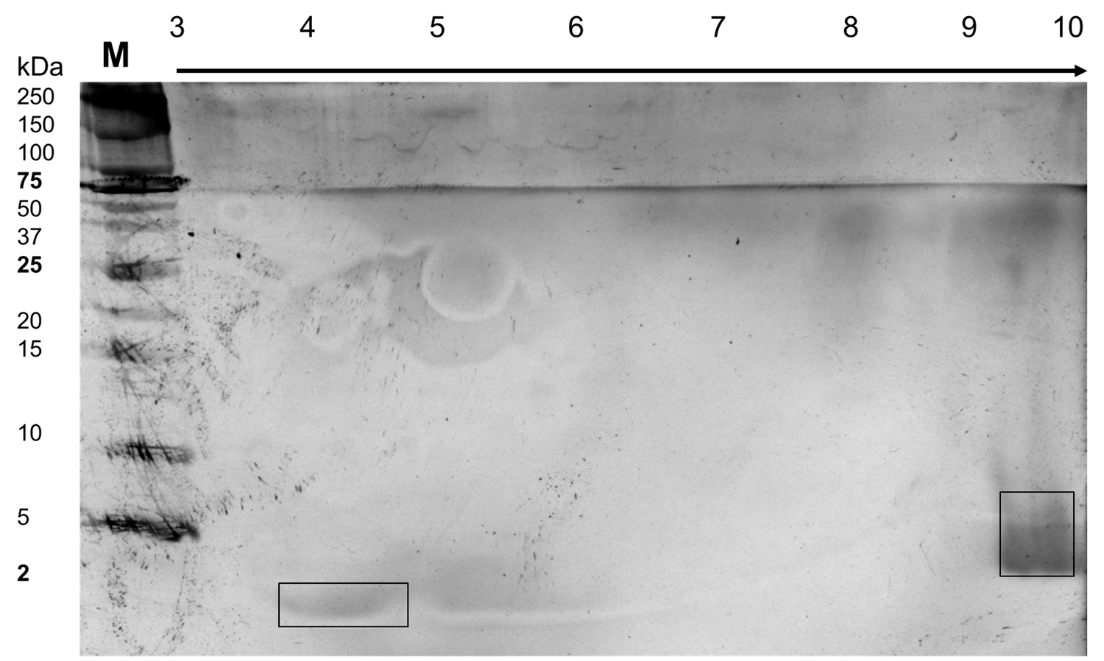
**

**Figure S8** – Isoelectric focusing of Gel4 in native conditions between pH3 and pH10. For 1D isoelectric focusing was used PROTEAN IEF system with ReadyStrips IPG pH3-10 (Biorad). For the 2D electrophoresis a Tris-Tricine gel used. Marker: Precision Plus Dual Xtra protein ladder (Biorad).

**
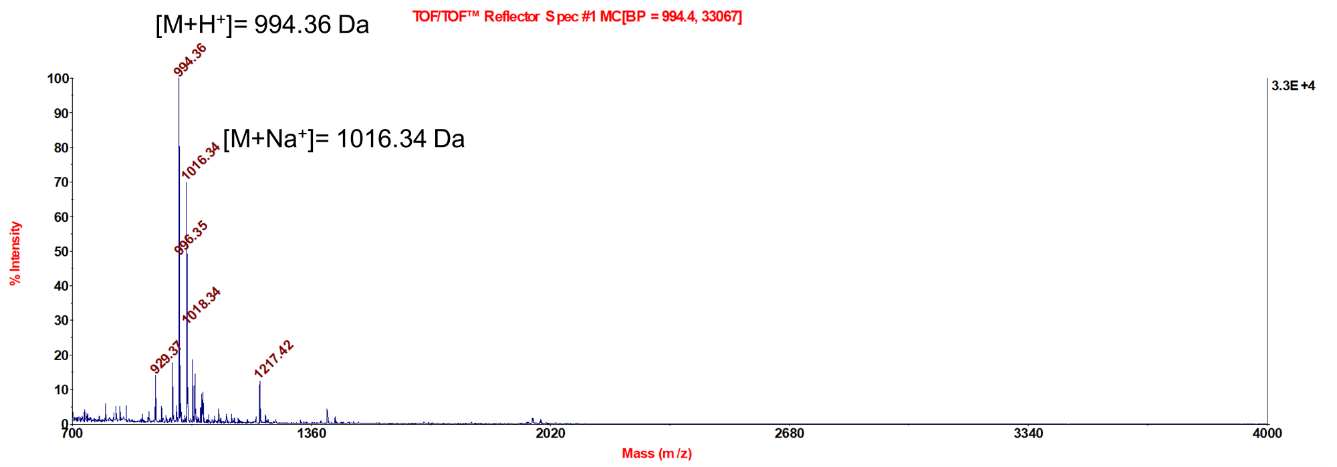
**

**Figure S9** – Mass spectra Maldi-TOF-TOF in positive mode of Gel4. A major peak with m/z 994.36Da is observed.

**Table S3 -** Solvent accessible surface area (SASA) values determined in Coarse-Grained Molecular Dynamics:

| **pH** | **SASA_initial_ (nm^2^)** | **SASA_final_ (nm^2^)** | **SASA_initial_ / SASA_final_ as a measure for the aggregation propensity** |
| --- | --- | --- | --- |
| 7 | 1947.437 | 332.022 | 5.865 |
| 4 | 1926.785 | 276.825 | 6.960 |

Note: SASA_initial_ - Total SASA after minimization; SASA_final_ - Total SASA after Molecular Dynamics


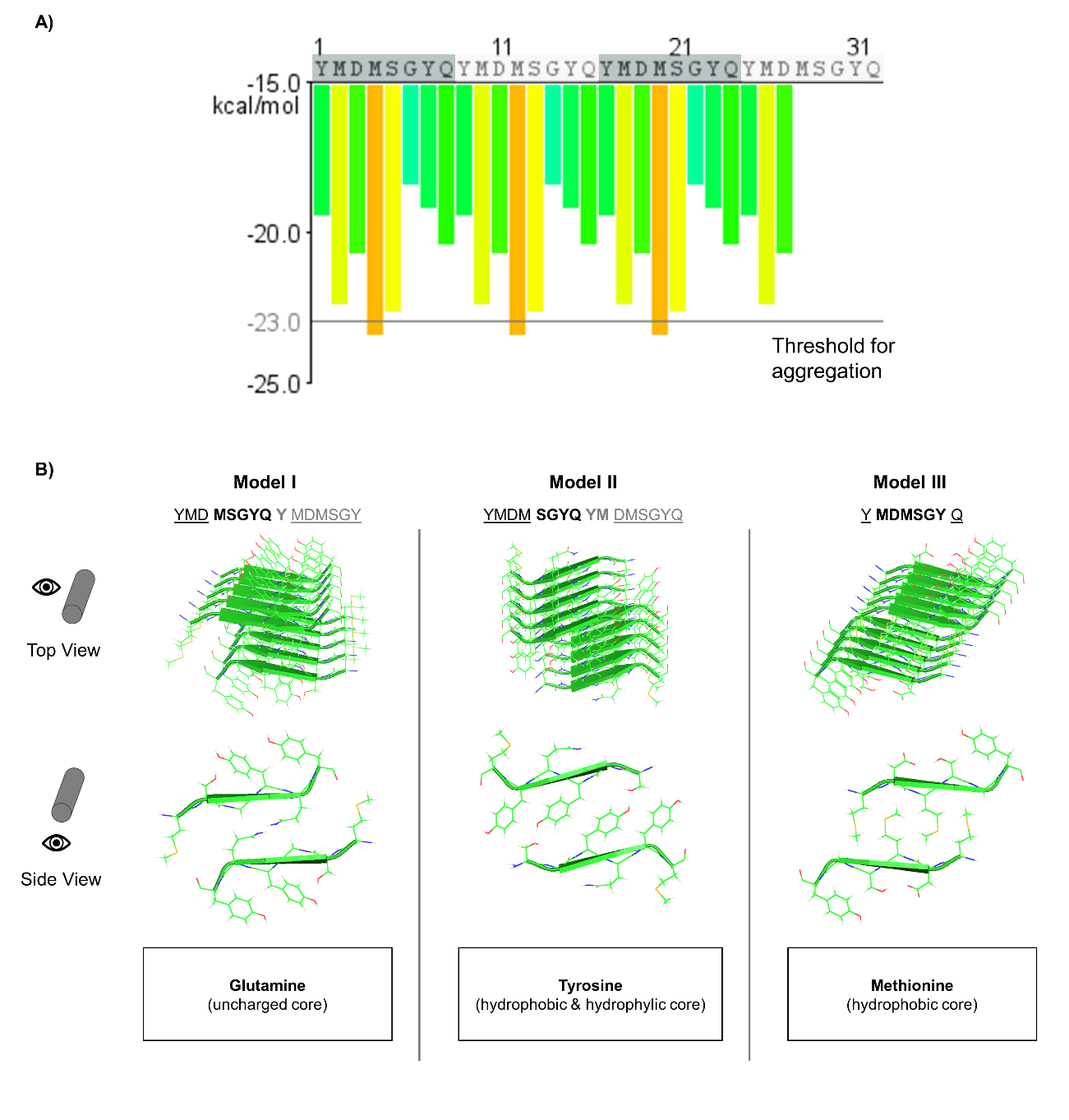


**Figure S10 –** Output of the submission from ZipperDB website. A) Graphical output with aggregation propensity (measured in energy, Kcal/mol) of the different amino acids. B) ZipperDB(Goldschmidt et al., 2010) top 3 outputs for protopeptide aggregation pattern: Model I, Model II and Model III. In Bold are identified the amino acids with highest propensity for aggregation by ZipperDB, in black and blue the amino acids from two different peptide chains and underlined the amino acids without aggregation propensity. Images designed in PyMOL(DeLano, 2002) visualization software.

**Table S4 –** ZipperDB peptide models top results.

| **Outputs Structure** | **Shape complementarity**  **(0 to 1)** | **Area buried at interface per layer (Å^2^)** | **Energy Score**  **(Kcal/mol)** |
| --- | --- | --- | --- |
| **MSGYQ Y** | 0.839 | 64 | 23.4 |
| **SGYQ YM** | 0.725 | 69 | 22.6 |
| **MDMSGY** | 0.904 | 83 | 22.4 |

Note: Underlined amino acids are in a second peptide chain.

**
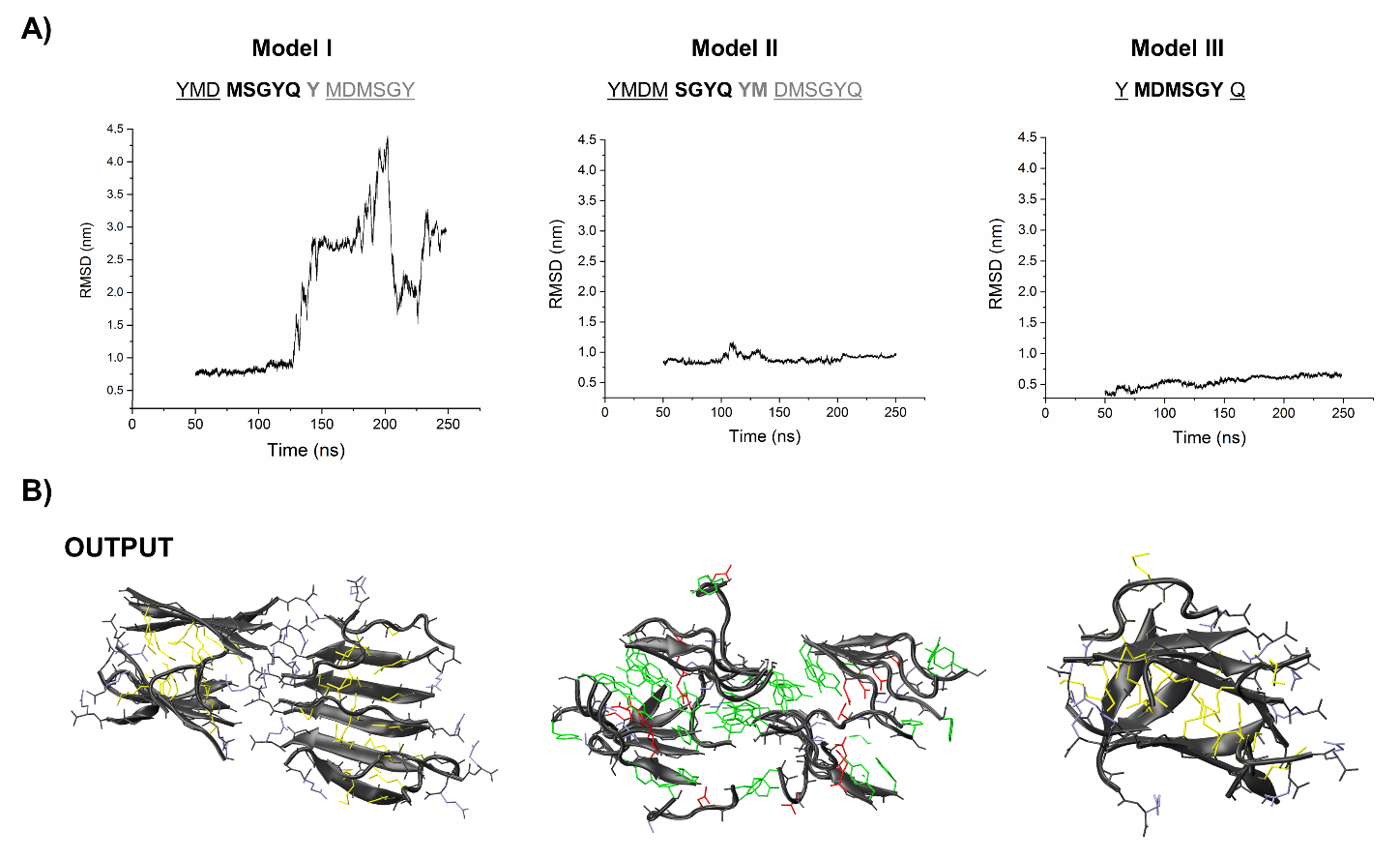
**

**Figure S11 –** All-Atom Molecular Dynamics simulations of protopeptide. A) Root Mean Square Deviation plots for bundle models. B) The output structures of 250 ns simulation for bundle models. Images designed in PyMOL(DeLano, 2002) visualization software.


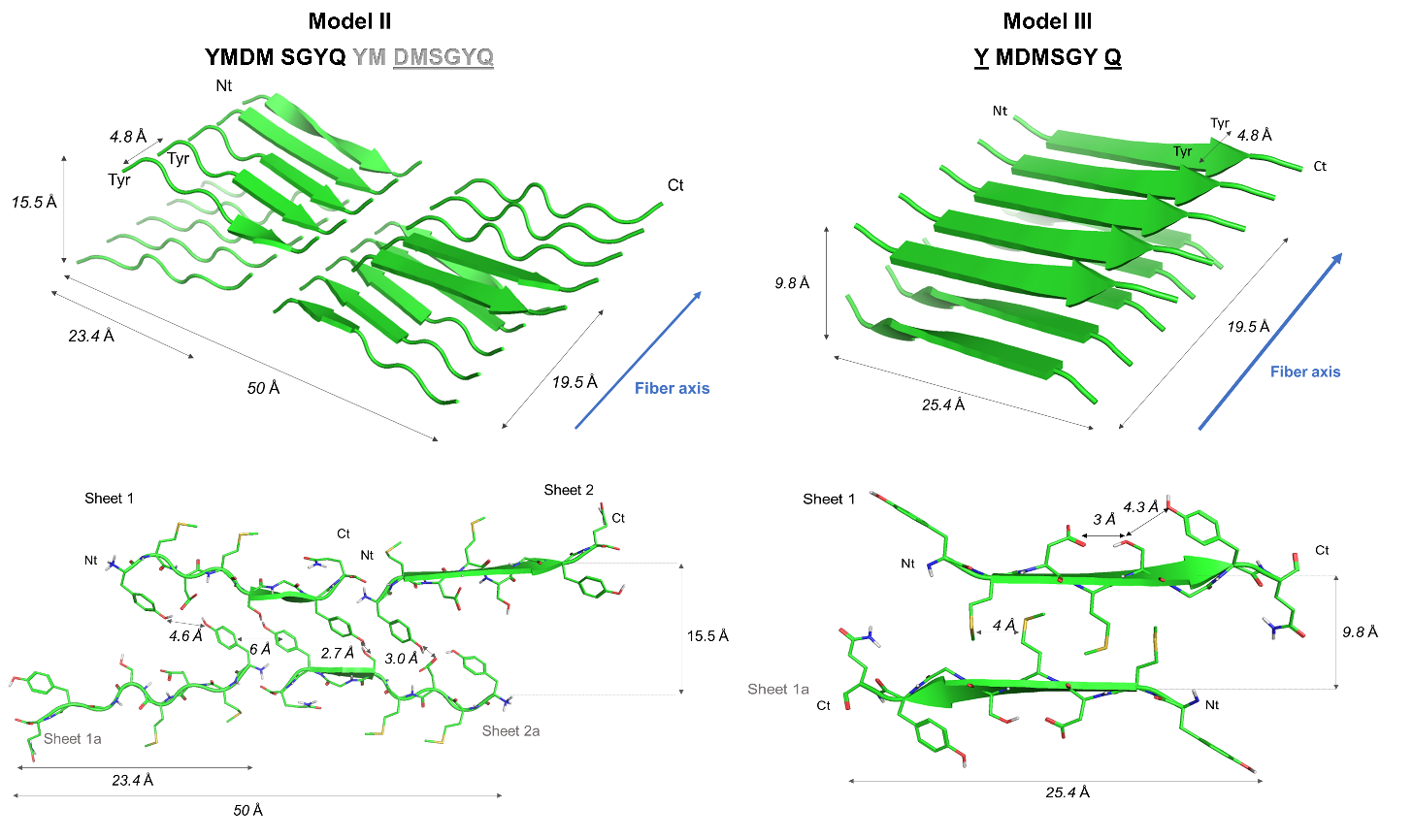


**Figure S12** – Analysis of critical distances for model II and III after All-Atom Molecular Dynamics simulations. Images designed in PyMOL(DeLano, 2002) visualization software.


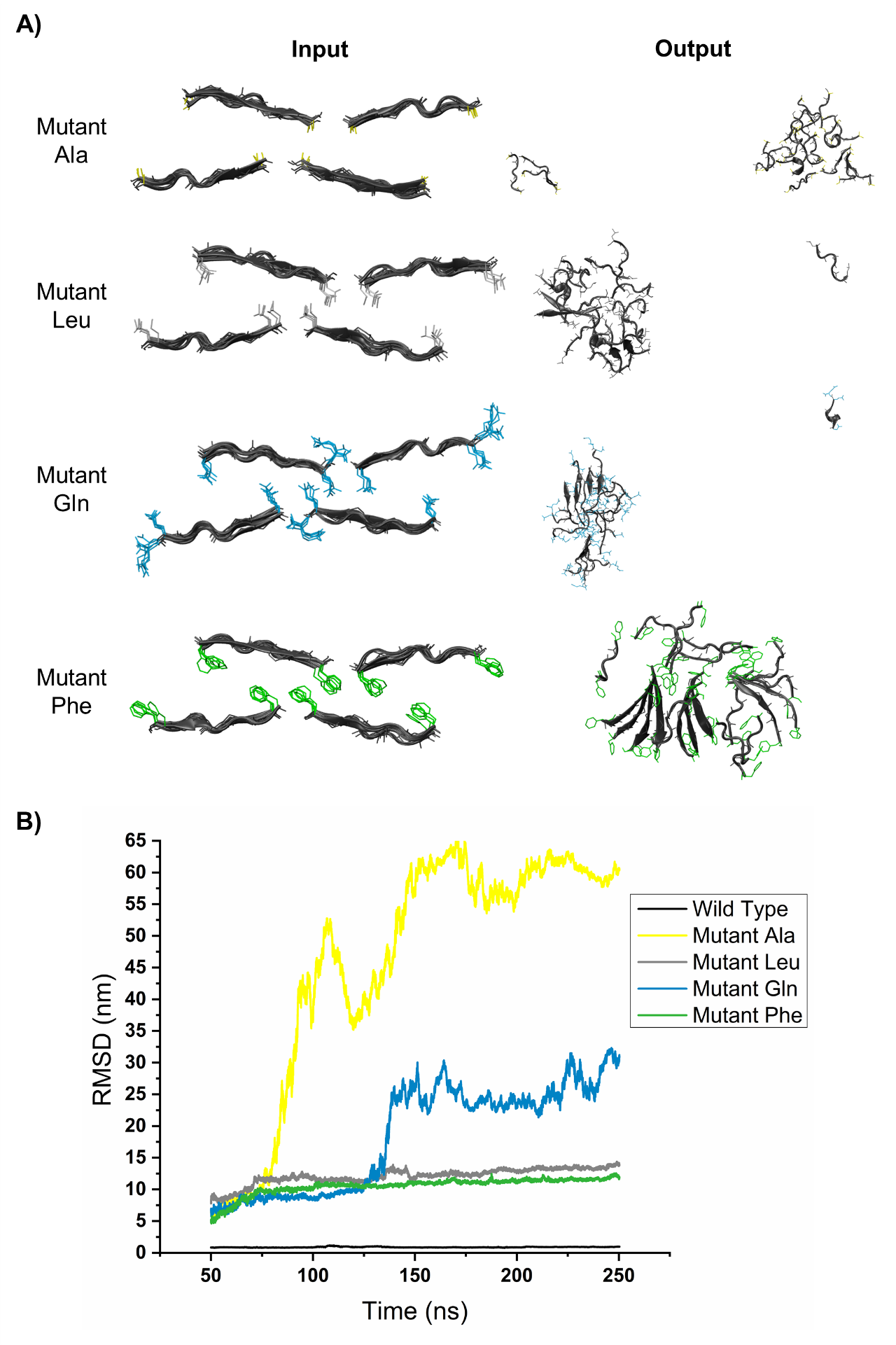


**Figure S13 -** All-Atom Molecular Dynamics simulations of protopeptide mutants. Model II was mutated in the Tyrosine (Wild Type, black) residues to: Ala (Mutant Ala, yellow), Leu (Mutant Leu, grey), Gln (Mutant Gln, blue) or Phe (Mutant Phe, green). A) The input and output structures of the mutants after 250 ns at 25 °C simulation. B) Comparison between Root Mean Square Deviation plots for Wild Type and mutants Ala, Leu, Gln and Phe. Images designed in PyMOL(DeLano, 2002) visualization software.

**Table S5** - Root Mean Square Deviation (RMSD) analysis for Wild Type protopeptide and mutants Ala, Leu, Gln and Phe after 250ns AA-MD simulation.

| Peptide | Sequence | RMSD after 250ns |
| --- | --- | --- |
| Wild Type | **Y**MDMSG**Y**Q | 0.98 |
| Mutant Ala | **A**MDMSG**A**Q | 60.40 |
| Mutant Leu | **L**MDMSG**L**Q | 13.87 |
| Mutant Gln | **Q**MDMSG**Q**Q | 30.86 |
| Mutant Phe | **F**MDMSG**F**Q | 11.85 |

**
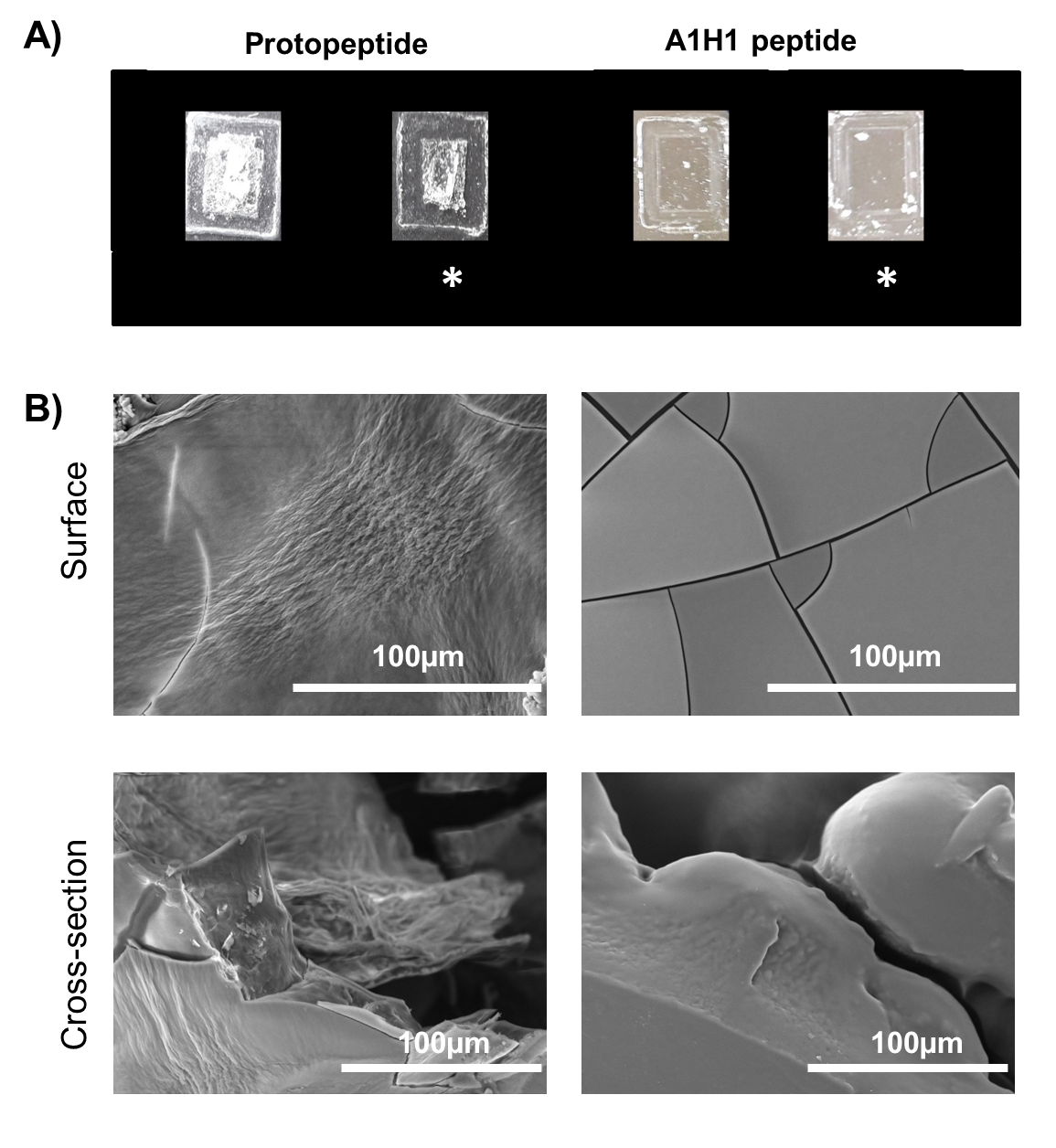
**

**Figure S14** – A) Peptide filmss produced for optical characterization. The (*) identifies the duplicate. B) SEM characterization of protopeptide and A1H1-based filmss.

**References:**

DeLano, W. (2002). Pymol: An open-source molecular graphics tool. *CCP4 Newsletter On Protein Crystallography*.

Goldschmidt, L., Teng, P. K., Riek, R., and Eisenberg, D. (2010). Identifying the amylome, proteins capable of forming amyloid-like fibrils. *Proc Natl Acad Sci U S A* 107, 3487–3492. doi: 10.1073/pnas.0915166107.

Guan, Z., Cai, T., Liu, Z., Dou, Y., Hu, X., Zhang, P., et al. (2017). Origin of the Reflectin Gene and Hierarchical Assembly of Its Protein. *Curr Biol* 27, 2833–2842. doi: 10.1016/j.cub.2017.07.061.

Nelson, D. L., Cox, M. M., and Hoskins, A. A. (2021). *Lenhinger Principles in Biochemistry*. 8th Editio. , eds. D. L. Nelson, M. M. Cox, and A. A. Hoskins Macmillan Learning.
